# Regulatory Role and Cytoprotective Effects of Exogenous Recombinant SELENOM under Ischemia-like Conditions and Glutamate Excitotoxicity in Cortical Cells In Vitro

**DOI:** 10.3390/biomedicines12081756

**Published:** 2024-08-05

**Authors:** Egor A. Turovsky, Egor Y. Plotnikov, Elena G. Varlamova

**Affiliations:** 1 Institute of Cell Biophysics of the Russian Academy of Sciences, Federal Research Center “Pushchino Scientific Center for Biological Research of the Russian Academy of Sciences”, 142290 Pushchino, Russia; 2 A.N. Belozersky Institute of Physico-Chemical Biology, Lomonosov Moscow State University, 119992 Moscow, Russia; plotnikov@belozersky.msu.ru; 3 V.I. Kulakov National Medical Research Center of Obstetrics, Gynecology and Perinatology, 117997 Moscow, Russia

**Keywords:** selenoprotein M, apoptosis, necrosis, cytosolic calcium, neuroprotection, astrocytes, neurons, ischemia-like conditions, reactive oxygen species, glutamate excitotoxicity

## Abstract

Despite the successes in the prevention and treatment of strokes, it is still necessary to search for effective cytoprotectors that can suppress the damaging factors of cerebral ischemia. Among the known neuroprotectors, there are a number of drugs with a protein nature. In the present study, we were able to obtain recombinant SELENOM, a resident of the endoplasmic reticulum that exhibits antioxidant properties in its structure and functions. The resulting SELENOM was tested in two brain injury (in vitro) models: under ischemia-like conditions (oxygen-glucose deprivation/reoxygenation, OGD/R) and glutamate excitotoxicity (GluTox). Using molecular biology methods, fluorescence microscopy, and immunocytochemistry, recombinant SELENOM was shown to dose-dependently suppress ROS production in cortical cells in toxic models, reduce the global increase in cytosolic calcium ([Ca^2+^]_i_), and suppress necrosis and late stages of apoptosis. Activation of SELENOM’s cytoprotective properties occurs due to its penetration into cortical cells through actin-dependent transport and activation of the Ca^2+^ signaling system. The use of SELENOM resulted in increased antioxidant protection of cortical cells and suppression of the proinflammatory factors and cytokines expression.

## 1. Introduction

Selenium is an essential micronutrient, and the recommended human intake is 55 µg/day for adult women and 70 µg/day for adult men [1]. Selenium deficiency in the body promotes the development of epilepsy in rodents and humans, while its supplementation in the diet suppresses epileptic seizures [2]. Selenium realizes its functions through selenoproteins and selenium-containing proteins, the disruption of whose expression leads to neurodegenerative diseases. Disturbances in the expression of selenoproteins and, consequently, the metabolism of selenium in the brain are associated with a number of neurodegenerative diseases such as epilepsy, Alzheimer’s disease, and Parkinson’s disease. At the same time, it has been shown that exogenous selenium in the form of nanoparticles or other compounds can alleviate the course of these diseases, primarily through suppression of oxidative stress, as well as the modulation of Ca^2+^ homeostasis and mitochondrial biogenesis, i.e., contributing to the maintenance of the energy balance of brain cells and their survival in the penumbra zone [3,4,5].

Selenoproteins are unique mammalian proteins because they contain residues of the selenium-containing amino acid selenocysteine and have a whole range of diverse functions—from antioxidant and immunomodulatory to the regulation of cell death and survival [6,7]. In humans, 25 selenoproteins have been identified, most of which are oxidoreductases involved in maintaining optimal cellular antioxidant status. Selenoproteins are found in the cytosol, mitochondria, and nucleus; a separate large group is expressed in the endoplasmic reticulum (ER), and these selenoproteins are called ER-resident selenoproteins. Of these 25 selenoproteins, 7 proteins are localized in the endoplasmic reticulum. The ER-resident selenoproteins are selenoprotein M (SELENOM), selenoprotein F (SELENOF), selenoprotein T (SELENOT), selenoprotein K (SELENOK), selenoprotein S (SELENOS), iodothyronine deiodinase 2 (DIO2), selenoprotein H (SELENOH), and selenoprotein N (SELENON) [8]. These proteins play an important role in regulating nerve cells redox status and protecting against oxidative stress. For all ER-resident selenoproteins, it has been established that disruption of their expression leads to activation of apoptosis [9].

Thioredoxin-interacting protein (TXNIP), also known as thioredoxin-binding protein 2, is a major mediator in the thioredoxin antioxidant system. TXNIP interacts with the CXXC motif of TXN, blocking its potential to scavenge ROS. This in turn increases the ROS levels in the cell and induces apoptosis [10]. However, their functions are not limited to antioxidant ones, and many functions of these proteins remain unexplored. It has been shown that ER-resident selenoproteins can participate in maintaining cellular Ca^2+^ homeostasis, regulating receptor-mediated neurotransmission and the GABAergic component of neurotransmission, protecting neurons from hyperexcitation and the toxic effects of glutamate during damage, and inhibiting ferroptosis in pathologies [11,12].

SELENOM is widely expressed in mammalian tissues—the heart, lung, kidney, stomach, small intestine, skin, testis, uterus, ovary, and brain, but is not expressed in the muscle and thymus [13,14]. In the brain, high levels of SELENOM expression are shown for the olfactory bulb, cortex, hippocampus, hypothalamus, brain stem, and cerebellum [14,15]. SELENOM in brain cells has been shown to participate in the regulation of the Ca^2+^ ion concentration in the ER and cytosol, as well as having an important role in antioxidant protection, since overexpression of SELENOM protects neurons from H_2_O_2_-induced ROS production. Interestingly, the highest level of SELENOM expression is found in GABAergic neurons [16]. Thioredoxin-interacting protein (TXNIP), also known as thioredoxin-binding protein 2, is a major mediator in the thioredoxin antioxidant system. TXNIP interacts with the CXXC motif of TXN, blocking its potential to scavenge ROS. This in turn increases the ROS levels in the cell and induces apoptosis [10]. The mechanisms of SELENOM involvement in neurotransmission have not been established, but there is some work with SELENOM knockouts. In a mouse model of Alzheimer’s disease, a correlation was established between the mutation of the presenilin-2 gene and a decrease in the expression of SELENOM [17]. SELENOM activates the ERK but not MAPK pathway involving p38 and JNK to attenuate alpha/gamma-secretase-mediated proteolysis and Tau phosphorylation to protect brain function [18]. The SELENOM knockout did not lead to disturbances at the level of brain morphology and development. However, mice with SELENOM deletion showed greater weight gain and fat accumulation compared to controls, which is caused by a tendency for the animals to have less energy expenditure and less mobility [15]. Also, SELENOM deletion may be involved in impaired synaptic plasticity, and this impairment was detected in male mice, since only they showed a lack of LTP responses. This sexual dimorphism is hypothesized to be related to the involvement of SELENOM in the regulation of insulin levels, and male SELENOM^−/−^-mice exhibited elevated fasting insulin levels compared to females [15].

The purpose of this investigation was to study the cytoprotective mechanisms of 24 h pre-incubation of cortical cells with recombinant SELENOM against the negative consequences of glutamate excitotoxicity and ischemia/reoxygenation. The premises of our work followed from the available data on the active participation of recombinant SELENOM in the processes occurring in the cortical cells. Overexpression of neuronal SELENOM has been shown to reduce H_2_O_2_-mediated intracellular Ca^2+^ flux. In contrast, knockout of the SELENOM gene promotes increased cytosolic Ca^2+^ levels, increased oxidative stress, and apoptosis [19]. It was also found that in neurons overexpressing presenilin 2 (PS2), Ca^2+^ efflux from the ER was associated with a decrease in the expression of this selenoprotein [17]. However, the mechanism underlying the regulation of Ca^2+^ homeostasis involving SELENOM remains unclear. In addition, SELENOM is known to be highly expressed in hypothalamic regions and is a positive regulator of leptin signaling and thioredoxin antioxidant activity in this brain region [20]. It has also been shown that SELENOM knockout in mice reduces synaptic plasticity; causes memory impairment and hyperglycemia; and disrupts glucose metabolism, which is essential for the formation and transmission of synapses in the brain [21].

## 2. Materials and Methods

### 2.1. Cloning and Site-Directed Mutagenesis

To obtain the recombinant cysteine-containing homolog of SELENOM, we isolated total RNA using RNA extract reagent (Evrogen, Moscow, Russia) according to the manufacturer’s protocol from human embryonic kidney (HEK 293), after which we performed a reverse transcription reaction using oligodT primers and a kit for the synthesis of the first-strand cDNA (Evrogen, Moscow, Russia). Next, we performed a routine cloning procedure for the open reading frame (ORF) of human SELENOM (hSELENOM) (NM_080430.3) into the pET23b(+) plasmid (Novagen, Merck Group, Darmstadt, Germany) using primer pairs 5′ TACAGAATTCATGAGCCTCCTGTTGCCTCCG 3′ and 5′ CGTCGACCTACAGGTCAGCGTGGTCCGAAG 3′. Since obtaining selenocysteine-containing SELENOM in preparative quantities is a very difficult task, we worked with the cysteine homologue of the native protein. To do this, we replaced selenocysteine located at position 69 with cysteine using overlapping primers 5′ GTAGAGACCTGCGGGGGATGTCAGCTGAACCGCC 3′ and 5′ GCTGACATCCCCCGCAGGTCTCTACCCG 3′.

### 2.2. Isolation and Purification of Recombinant hSELENOM

Isolation and purification of recombinant SELENOM from bacterial cells was carried out under native conditions. Due to the presence of a polyhistidine tag at the C-terminus of recombinant SELENOM, its purification was carried out using affinity chromatography on nickel agarose (Thermo Fisher Scientific, Waltham, MA, USA) (Figure S1). The procedures described in detail are presented in our work [22].

### 2.3. Western Blot Analysis

To perform Western blot analysis, cortical cells were lysed in a buffer containing 100 mM Tris-HCl (pH 8.0), 0.15 mM NaCl, 1 mM EDTA, and 1 mM PMSF, at 4 °C. After that, electrophoresis of the studied lysates was carried out in 12.5% PAAG, 50 μg of total protein was added to each well, and then the mixture was transferred to a nitrocellulose membrane. The membranes were blocked in 5% dry non-fat milk for 3 h at room temperature, after which the membranes were washed and incubated with primary antibodies against polyhistidine tag (Cat. No. MA1-21315, Thermo Fisher Scientific, Waltham, MA, USA) and against GAPDH (Cat. No. MA5-15738, Thermo Fisher Scientific, Waltham, MA, USA) overnight at 4 °C. Next, the membranes were thoroughly washed and incubated with secondary antibodies labeled with horseradish peroxidase (Cat. No. 31402, Thermo Fisher Scientific, Waltham, MA, USA).

### 2.4. Preparation of Mixed Neuroglial Cell Cultures

All animal experiments were performed in accordance with the experimental protocols approved by the Bioethics Committee of the Institute of Cell Biophysics (Approval ID: 1/092022, date: 8 September 2022). Experiments were performed in accordance with the Rules of Laboratory Practice for the Care and Use of Laboratory Animals and Directive 2010/63 EU of the European Parliament on the protection of animals used for scientific purposes. Cell cultures from mouse cerebral cortex were prepared as described in detail previously [23]. Briefly, 0–1-day-old pups were euthanized and decapitated. The extracted tissue was washed with Mg^2+^- and Ca^2+^-free Versene solution and minced with scissors. Then, the tissue fragments were digested with 1% trypsin solution for 10 min at 37 °C and washed two times with cold Neurobasal-A medium. Trypsinized tissue was gently triturated with a pipette, and the debris was then carefully removed with a pipette tip. The obtained cell suspension was seeded on polyethyleneimine-coated glass coverslips and grew for 8 days in vitro in the cell culture medium composed of Neurobasal-A medium, supplement B-27 (2%), and 0.5 mM glutamine. The density of plated cells was 15,000 cells/cm^2^.

### 2.5. Cultivation of Mouse Astrocytes

Astroglial cell cultures were isolated from the brains of 1–2-day-old mice according to the modified protocol of McCarthy and de Vellis [24,25]. The brains were extracted, the cerebral cortex separated, the meninges removed, and the tissue minced and then incubated in 0.05% trypsin-EDTA solution at 37 °C for 30 min. After enzymatic digestion, tissues were washed twice in PBS and then dissociated with a glass pasteur pipette in culture medium consisting of DMEM (PanEco, Moscow, Russia), 1 g/L D-glucose, and 10% FBS (Biosera, Kansas City, MO, USA) with the addition of 2 mM glutamine (PanEco, Moscow, Russia). The cell suspension was transferred on ventilated culture vials (Costar, Washington, DC, USA) pre-coated with poly-D-lysine (10 µg/mL). Cells were cultured at 37 °C and 5% CO_2_. After 5–6 days, cultures were shaken on an orbital shaker at 200 rpm for 16 h to detach and remove microglia. After 10–20 days, the in vitro astrocytes were used for experiments. Mouse astrocytes cultured in 60 mm diameter Petri dishes were washed with warm (37 °C) versene and then treated with trypsin. After 3–5 min, the cell suspension was removed from the Petri dishes and centrifuged at 2500 rpm for 3 min. The obtained cell suspension was seeded on polylysine-coated glass cover slips or 96-well plates and grown for 5 days in vitro in the cell culture medium composed of DMEM with 10% FBS and 2 mM glutamine. The density of plated cells was 25.000 cells/cm^2^.

### 2.6. The Technique for Simulation of Ischemia-like Conditions

Ischemia-like conditions (oxygen-glucose deprivation, OGD) were obtained by omitting glucose (HBSS medium without glucose) and by displacement of dissolved oxygen with argon in the leak-proof system [26]. The level of oxygen in the medium was measured using a Clark electrode. Oxygen tensions reached values of 30–40 mm Hg or less within 20 min after the beginning of displacement. Ischemia-like conditions lasting for ~40 min were created by supplying the oxygen-glucose deprivation (OGD) medium into the chamber with cultured cortical cells. Constant argon feed into the experimental chamber was used to prevent the contact of the OGD medium with the atmospheric air.

### 2.7. Simulation of Glutamate Excitotoxicity

To create acute (~40–50 min) conditions for the excitotoxic action of glutamate (GluTox), 100 mM glutamate was added to the cells in a magnesium-free medium with the addition of 20 µM glycine. The effects of GluTox on neurons and astrocytes were evaluated by measuring the amplitude, shape, and rate of cell calcium response and assessment of cell viability before and after GluTox conditions. To induce long-term GluTox and reveal the role of SELENOM on apoptosis processes, 300 µM glutamate plus 20 µM glycine was added to the culture medium for 24 h, while magnesium ions were present in the culture medium [27].

### 2.8. Assessment of Cell Viability

Propidium iodide (1 µM) was used to evaluate the number of dead cells in the cell cultures before and after OGD. The cells were stained for 5 min with the probes diluted in HBSS and then rinsed with HBSS. Fluorescence of the probes was detected with an inverted fluorescent microscope Zeiss Axio Observer Z1 using Filter Set 20. Cell death induced by OGD or GluTox was assessed by propidium iodide staining (PI, 1 µM) before and after the exposures in the same microscopic field [28]. Five different areas of each cell culture were analyzed. Each experimental group consisted of three cell cultures from different passages.

Hoechst 33342 (2 µM) and propidium iodide (1 µM) were used to evaluate the number of dead cells in the cell cultures before and after 2 h OGD and 24 h reoxygenation or 24 h GluTox. The cells were stained for 5 min with the probes diluted in HBSS and then rinsed with HBSS. Fluorescence of the probes was detected with an inverted fluorescent microscope Zeiss Axio Observer Z1 using Filter Set 01 and Filter Set 20 (Carl Zeiss, Oberkochen, Germany). Discrimination of early and late apoptotic cells was performed according to the previously described method [28]. Five different areas of each cell culture were analyzed. Each experimental group consisted of three cell cultures from different passages.

### 2.9. Fluorescent Ca^2+^ Measurements

To detect the changes in [Ca^2+^]_i_, cell cultures were loaded with Fura-2 (4 µM; 40 min incubation; 37 °C). The cells were stained with the probe dissolved in Hank‘s balanced salt solution (HBSS) composed of (mM) 156 NaCl, 3 KCl, 2 MgSO_4_, 1.25 KH_2_PO_4_, 2 CaCl_2_, 10 glucose, and 10 HEPES (pH 7.4). To measure [Ca^2+^]_i_, we used the system based on an inverted motorized microscope Leica DMI6000B with a high-speed monochrome CCD-camera HAMAMATSU C9100. For excitation and registration of Fura-2 fluorescence, we used the FU-2 filter set (Leica Microsystems, Wetzlar, Germany) with excitation filters BP340/30 and BP387/15, beam splitter FT-410, and emission filter BP510/84. The Illuminator Leica EL6000 with a high-pressure mercury lamp was used as a source of excitation light. To distinguish neurons and astrocytes, we used short-term applications of 35 mM KCl before the main experiments. Briefly, KCl induces depolarization of excitable cells, which contain a wide range of voltage-gated cation channels. KCl-induced depolarization promotes the opening of voltage-gated calcium channels in neurons (predominantly L-type channels). The conductivity and density of cation channels in astrocytes are insufficient to evoke high-amplitude Ca^2+^ response to KCl application. All the Ca^2+^ signals are presented as 340/380 ratio of Fura-2 fluorescence.

### 2.10. Extraction of RNA

Isolation of RNA from cell cultures was performed using a reagent for the isolation of total RNA-Extract tRNA reagent (Evrogen, Moscow, Russia) containing a solution of phenol and guanidine isothiocyanate. The reagent was added to a Petri dish with a cell monolayer at the rate of 1 mL per 10 cm^2^ of the surface, after which total RNA was isolated according to the manufacturer’s protocol. The quality of isolation RNA was checked by electrophoresis in 1% agarose gel, as well as on a spectrophotometer at a wavelength of 260 nm. To prevent contamination of genomic DNA, RNA samples were treated with DNase I at 37 °C for 1 h, after which the enzyme was inactivated by adding 50 mM EDTA to the mixture and heating to 60 °C for 10 min.

The reverse transcription reaction was carried out according to the protocol and using a kit for the synthesis of the first-strand cDNA (Evrogen, Moscow, Russia) containing reverse transcriptase MMLV. The reaction was carried out in the presence of oligo(dT) primers. The content of total RNA (1–2 µg) used in reverse transcription reactions was controlled by parallel amplification using primers specific to the reference gene.

### 2.11. Quantitative Real-Time Polymerase Chain Reaction (RT-qPCR)

Each PCR was performed in a 25 μL mixture composed of 5 μL of qPCRmix-HS SYBR (Evrogen, Moscow, Russia), 1 μL (0.2 μM) of the primer solution, 17 μL water (RNase-free), and 1 μL cDNA. A Dtlite Real-Time PCR System (DNA-technology, Moscow, Russia) was used for amplification. The amplification process consisted of the initial 5 min denaturation at 95 °C, 40 cycles of 30 s denaturation at 95 °C, 20 s annealing at 60–62 °C, and 20 s extension step at 72 °C. The final extension was performed for 10 min at 72 °C. All the sequences were designed with FAST PCR 5.4 and NCBI Primer-BLAST software (https://www.ncbi.nlm.nih.gov/tools/primer-blast/primertool.cgi, accessed on 6 July 2023). The data were analyzed with Dtlite software (https://dna-technology.com/sites/default/files/dtprime_dtlite_v06_part_2.pdf, accessed on 6 July 2023; DNA-technology, Moscow, Russia). The expression of the studied genes was normalized to the gene encoding glyceraldehyde 3-phosphate dehydrogenase (GAPDH). Data were analyzed using Livak’s method.

### 2.12. Measurement of ROS Production in Cortical Astrocytes

For simultaneous recordings of changes in mitochondrial or cytosolic ROS production, cortical astrocyte monocultures were loaded with MitoSOX Red (mitochondrial ROS indicator, 5 µM, 15 min incubation; 37 °C) or H_2_DCF-DA (mainly cytosolic ROS indicator; 10 µM, 20 min incubation; 37 °C). Cells were stained with the probes dissolved in Hank’s balanced salt solution (HBSS) composed of (mM) 156 NaCl, 3 KCl, 2 MgSO_4_, 1.25 KH_2_PO_4_, 2 CaCl_2_, 10 glucose, and 10 HEPES (pH 7.4). After incubation with the dyes, cells were washed three times before the experiment. To measure the ROS generation, we used the system based on the inverted motorized microscope Leica DMI6000B with a high-speed monochrome CCD-camera HAMAMATSU C9100 and a high-speed light filter replacing system Leica’s Ultra-Fast Filter Wheels with a replacing time of 10–30 ms. For excitation of H_2_DCF-DA and MitoSOX Red, we used the L5 filter set (Leica Microsystems, Wetzlar, Germany) with an excitation filter of BP480/40, dichroic mirror of 505, and emission filter of 527/30. The shape and speed of ROS production rates under SELENOM or H_2_O_2_ application were determined.

To register the total ROS production to reveal the concentration effects of SELENOM, the cortical astrocytes were grown in 96-well plates for 5 days. Next, various concentrations of the SELENOM were added to the culture medium for 24 h, and after that, the cells were washed and loaded with H_2_DCF-DA (20 µM, 30 min incubation; 37 °C). In the acute experiment, cells were loaded with the H_2_DCF-DA probe (20 µM, 30 min incubation; 37 °C), various concentrations of SELENOM were applied, and ROS production was recorded for 3 h. DCF fluorescence was recorded using an automated multiplate reader with SparkControl software (Spark™ 10M multimode microplate reader; Tecan Trading AG, Männedorf, Switzerland). After detecting the base level of fluorescence intensity, H_2_O_2_ at a concentration of 100 µM was added to initiate the ROS production. To avoid photo destruction of the probe and to avoid the photodynamic ROS production, DCF fluorescence was recorded once every 5 min. ImageJ, Origin 8.5, and Prism GraphPad software (GraphPad Software, RRID: SCR_002798) were used in order to analyze data, create graphs, and perform statistical tests. All values are given as mean ± SEM.

### 2.13. Immunocytochemical Method

In order to detect GFAP, NeuN, and Hexa Histidine in cells, we used an immunocytochemical assay. The cells were fixed with 4% paraformaldehyde + 0.25% glutaraldehyde in PBS for 20 min and washed three times with ice-cold PBS for 5 min. Glutaraldehyde was added into the fixative solution to minimize washing of antibodies from cells during permeabilization. To permeabilize cells, we used 0.1% Triton X-100 solution for 15 min. Fixed cells were incubated in 10% donkey serum for 30 min at room temperature to block nonspecific antibody binding sites. The cells were then incubated with primary antibodies against the investigated proteins for 12 h at 4 °C. The fixed cells were subsequently washed with PBS (3 times for 5 min) and probed with secondary antibodies conjugated with fluorescent label. We used purified rabbit polyclonal antibody to glial fibrillary acidic protein (GFAP) (Sigma-Aldrich, Burlington, MA, USA, AB5804), rabbit polyclonal Anti-NeuN antibody (Abcam, Cambridge, UK, RRID: AB_10711153), mouse monoclonal antibody to Hexa Histidine (H6) (Cloud-Clone Corp., Katy, TX 77494, USA, MAX656Ge22), donkey polyclonal secondary antibody to rabbit IgG (H+L) (Alexa Fluor-647) (Jackson ImmunoResearch Europe LTD, Cambridge, UK, RRID: AB_2492288), and donkey polyclonal secondary antibody to mouse IgG-H&L (Alexa Fluor-594) (Abcam, Cambridge, UK, RRID: AB_2732073). Dilutions of primary and secondary antibodies were performed according to the manufacturer’s recommendations for immunocytochemical staining. The fluorescence of antibodies was visualized with a Leica TCS inverted confocal microscope SP5 (Leica, Wetzlar, Germany). Registration of the secondary antibodies’ fluorescence for the control and experimental groups of cell cultures was carried out at the same microscope setting. Fluorescence analysis was performed in Image J 2002 software (Wayne Rasband, Kensington, MD, USA, RRID: SCR_003070) using the Analyze particles and Time series analyzer plugins.

### 2.14. Statistical Analysis

All presented data were obtained from at least three cell cultures. All values are given as mean ± standard error (SEM) or as individual cellular signals in experiments. We used the Shapiro–Wilk test to determine the normality of the distribution. Statistical analyses were performed by Student’s *t*-test, nonparametric Mann–Whitney U test, or by non-parametric multiple comparison by the Kruskal–Wallis test. Differences are significant at * *p* < 0.05, ** *p* < 0.01, *** *p* < 0.001; n/s—data not significant (*p* > 0.05). MS Excel (Microsoft Office 2016, Redmond, Washington, DC, USA), ImageJ (developed by LOCI at the University of Wisconsin, Madison, WI, USA, available at https://imagej.nih.gov/ij/download.html, accessed on 18 May 2023), Origin 2016 (OriginLab, Northampton, MA, USA), and Prism GraphPad 7 (GraphPad Software, RRID: SCR_002798) software were used for data and statistical analysis.

## 3. Results

### 3.1. Exogenous SELENOM Protected Cortical Cells from Death under Excitotoxic Concentrations of Glutamate (GluTox) or Ischemia-like Conditions (OGD/R) In Vitro

**Figure 1:**
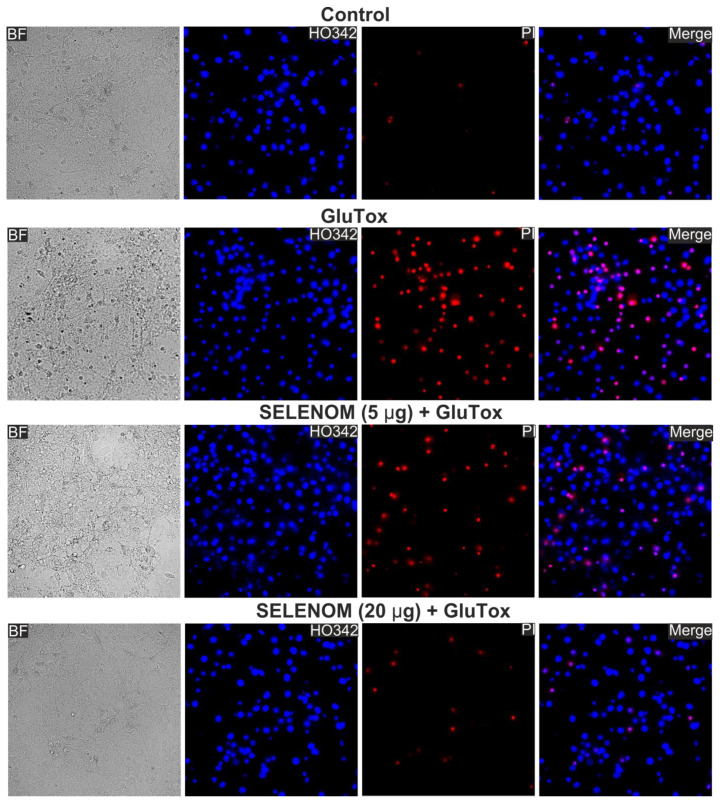
Effect of 24 h pre-incubation of neuroglial cortical cultures with 5 or 20 μg/mL exogenous SELENOM on GluTox-induced cell death. Double staining of cells with Hoechst 33342 (HO342) and propidium iodide (PI). GluTox—induction of the excitotoxic effect of glutamate (300 µM glutamate for 24 h) without prior incubation with SELENOM. SELENOM 5 µg and SELENOM 20 µg—24 h pre-incubation of cells with SELENOM and then 300 µM glutamate (GluTox) was added to the culture medium for 24 h.

**Figure 2:**
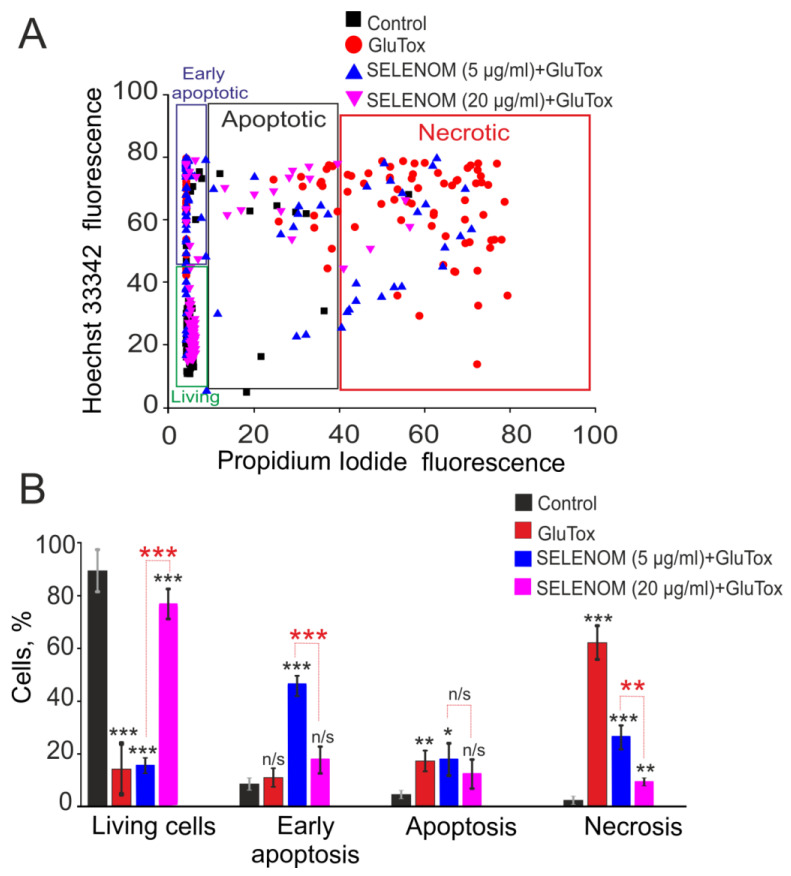
Effect of 24 h pre-incubation of cortical cells with various concentrations of exogenous SELENOM on GluTox-induced cell death. (**A**) Cytogram demonstrating the viability of cortical cells after 24 h of exposure to GluTox depending on the concentration of SELENOM. *X*-axis—PI fluorescence intensity. The *Y*-axis is the fluorescence intensity of Hoechst 33342. Cells were stained with probes 24 h after GluTox. (**B**) Effect of 24 h pre-incubation with 5 or 20 μg of SELENOM on the induction of necrosis and apoptosis. Black asterisks indicate the differences between the experimental groups comparable to the Control group. Differences between experimental groups are marked with asterisks of different colors. n/s—data not significant (*p* > 0.05), *** *p* < 0.001, ** *p* < 0.01, and * *p* < 0.05. Cell images are presented in Figure 1.

Double staining of cells with propidium iodide (PI) and Hoeschst 33342 (HO342) allows for the detection of cells at different stages of apoptosis and necrosis. After 24 h of exposure to an excitotoxic glutamate concentration (GluTox, 300 μM glutamate plus 20 μM glycine), 54 ± 11% of cells died by necrosis (registered by PI fluorescence), 19 ± 8% of cells in the late and 12 ± 6% of cells at the early stage of apoptosis, and no more than 15 ± 9% of cells remained viable (Figure 1—GluTox; Figure 2A,B—GluTox). Without exposure to GluTox, 93 ± 5% of cells remained viable, and apoptosis was detected in 10–14% of cells (Figure 1—Control; Figure 2A,B—Control). The use of exogenous SELENOM showed the inhibition of cell death after 24 h exposure with GluTox, recorded by a decrease the cells number with PI fluorescence and a decrease level of HO342 fluorescence (Figure 1—SELENOM + GluTox; Figure 2A,B—SELENOM + GluTox). Pre-incubation of neuroglial cortical cultures with 5 μg/mL SELENOM reduced the number of cells with early apoptosis to 48 ± 7% and late apoptosis to 18 ± 4%, and the number of necrotic cells decreased to 27 ± 4% (Figure 1—SELENOM (5 μg) + GluTox; Figure 2A,B—SELENOM (5 μg) + GluTox). Increasing the concentration of exogenous SELENOM to 20 μg/mL led to an even more pronounced decrease in the number of cells with early apoptosis (18 ± 5%), and the percentage of cells in the late stages of apoptosis did not change significantly when compared with 5 μg/mL SELENOM. In this case, the number of cells with GluTox-induced necrosis was 18 ± 6% (Figure 1—SELENOM (20 μg) + GluTox; Figure 2A,B—SELENOM (20 μg) + GluTox).

**Figure 3:**
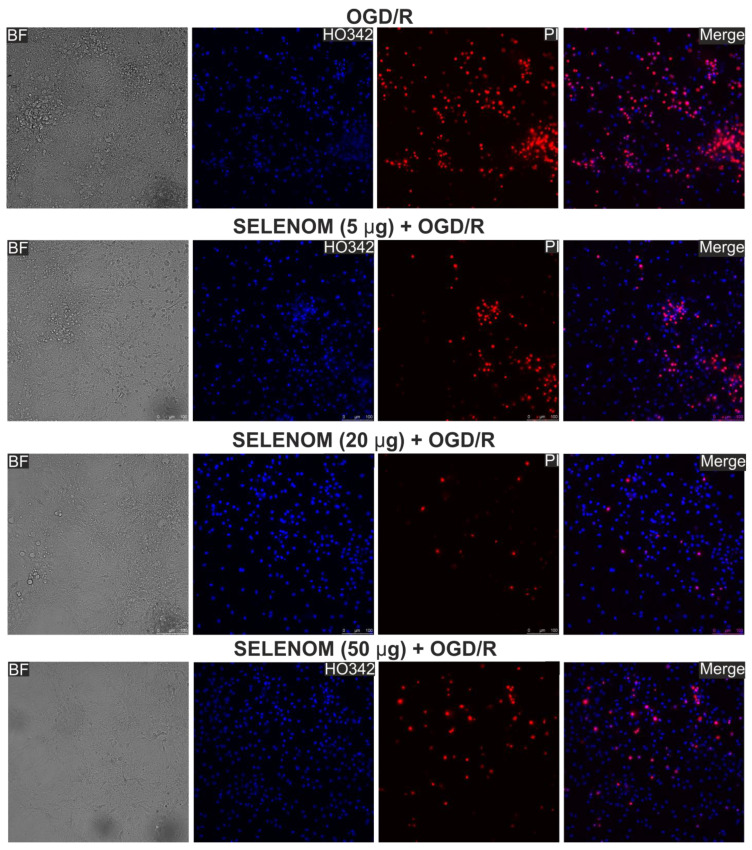
Effect of 24 h pre-incubation of neuroglial cortical cultures with 5, 20, or 50 μg/mL exogenous SELENOM on OGD/R-induced cell death. Double staining of cells with Hoechst 33342 (HO342) and propidium iodide (PI). OGD/R—induction of ischemia-like conditions (2 h) and reoxygenation for 24 h without pre-incubation with SELENOM. SELENOM 5 µg + OGD/R, SELENOM 20 µg + OGD/R, and SELENOM 50 µg + OGD/R—24 h pre-incubation of cells with SELENOM followed by creation of OGD/R.

**Figure 4:**
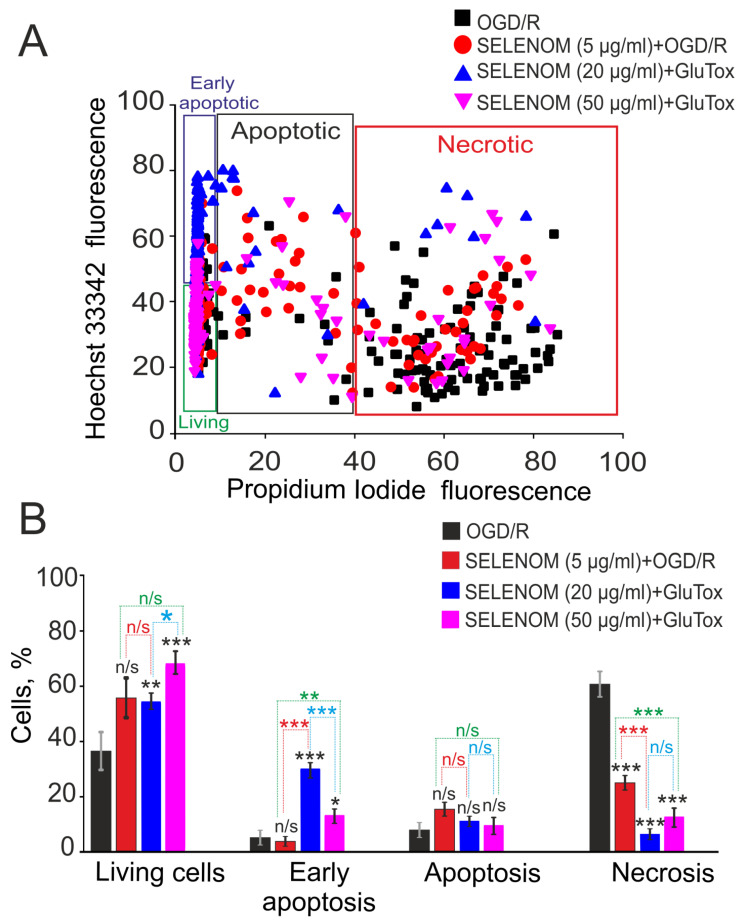
Effect of 24 h pre-incubation of cortical cells with various concentrations of exogenous SELENOM on OGD/R-induced cell death. (**A**) Cytogram demonstrating the viability of cortical cells after 24 h of exposure to OGD/R depending on the concentration of SELENOM. *X*-axis—PI fluorescence intensity. The *Y*-axis is the fluorescence intensity of Hoechst 33342. Cells were stained with probes 24 h after OGD/R. (**B**) Effect of 24 h pre-incubation with 5, 20, or 50 μg/mL SELENOM on the induction of necrosis and apoptosis in OGD/R. Designations: OGD/R—induction of ischemia-like conditions (2 h of oxygen-glucose deprivation) and reoxygenation for 24 h without prior incubation with SELENOM. SELENOM 5 µg + OGD/R, SELENOM 20 µg + OGD/R, and SELENOM 50 µg + OGD/R—24 h pre-incubation of cells with SELENOM followed by creation of OGD/R. Results are presented as mean ± SEM. Black asterisks indicate the differences between the experimental groups comparable to the OGD group. Differences between experimental groups are marked with asterisks of different colors. n/s—data not significant (*p* > 0.05), *** *p* < 0.001, ** *p* < 0.01, and * *p* < 0.05. Cell images are presented in Figure 3.

In the ischemia/reoxygenation in vitro model, exogenous recombinant SELENOM also showed high cytoprotective potential. Ischemia-like conditions (oxygen-glucose deprivation, OGD) for 2 h were followed by 24 h of reoxygenation, and then the cortical cells were stained with propidium iodide (PI) and Hoeschst 33342 (HO342). After OGD/R, 63 ± 11% of cells died as a result of necrosis, and apoptosis was detected in 10% of cells (4% early apoptosis and 6% late stages) (Figure 3—OGD/R; Figure 4A,B—OGD/R). Pre-incubation of cortical cells with 5, 20, or 50 μg/mL recombinant SELENOM resulted in decreases in necrotic cell death after OGD/R to 23%, 5%, and 14%, respectively, with increasing concentrations of SELENOM (Figure 3—SELENOM + OGD/R; Figure 4A,B—SELENOM + OGD/R). At the early stage of apoptosis after incubation with 5 μg/mL SELENOM, 3% of cells were recorded; increasing the concentration of exogenous SELENOM to 20 μg/mL and 50 μg/mL led to a slight increase in the number of cells with early apoptosis to 28% and 12%, respectively (Figure 4A,B). Conversely, with an increase in the concentration of exogenous SELENOM, a decrease in OGD/R-induced late stages of apoptosis occurred—under the influence of 5 μg/mL SELENOM, apoptosis was recorded in 16% of cells; at 20 μg/mL in 10% of cells; and after exposure with 50 μg/mL SELENOM, OGD/R-induced apoptosis was detected in 9% of cells (Figure 4A,B).

**Figure 5:**
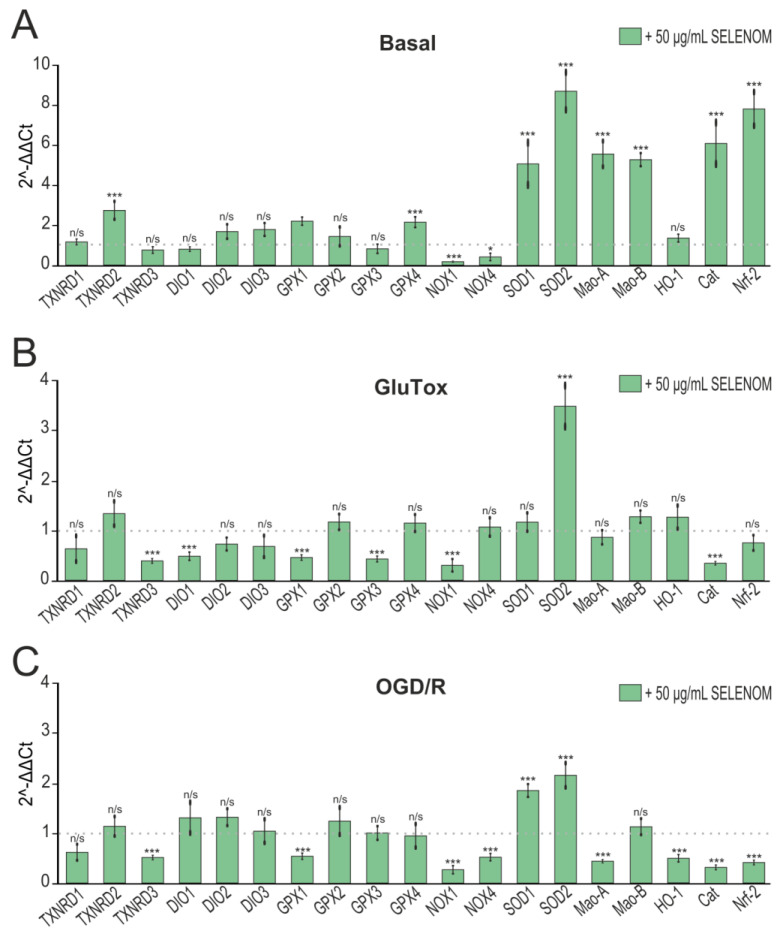
Effect of 24 hours’ pre-incubation of neuroglial cortical cultures with 5, 20, or 50 μg/mL exogenous SELENOM on OGD/R-induced cell death. Double staining of cells with Hoechst 33342 (HO342) and propidium iodide (PI). OGD/R—induction of ischemia-like conditions (2 h) and reoxygenation for 24 h without pre-incubation with SELENOM. SELENOM 5 µg + OGD/R, SELENOM 20 µg + OGD/R, and SELENOM 50 µg + OGD/R—24 h pre-incubation of cells with SELENOM followed by creation of OGD/R. Effect of 24 h pre-incubation of cortical neuroglial cultures with 50 μg/mL exogenous SELENOM on basal (**A**), GluTox-induced (**B**), and OGD/R-induced (**C**) expression of genes encoding proteins involved in redox status regulation. The dotted line indicates protein expression in cortical cells treated with solvent buffer for panel (**A**), in cells after 24 h of GluTox exposure without pre-incubation with SELENOM for panel (**B**), and ischemia-like conditions (OGD/R) without pre-incubation with SELENOM for panel (**C**). Results are presented as mean ± SEM. Black asterisks indicate the differences between the experimental groups comparable to the Buffer-treated group (**A**), GluTox-treated group (**B**), and OGD/R-treated group (**C**) without pre-incubation with SELENOM. n/s—data not significant (*p* > 0.05), *** *p* < 0.001, * *p* < 0.05.

Long-term exposure to biologically active substances, including proteins, involves changes in gene expression. Analysis of the genes expression encoding redox status proteins after 24 h pre-incubation of cortical cells with 50 μg/mL of recombinant SELENOM showed a significant increase in the expression of the genes TXNRD2 (thioredoxin reductase), GPX4 (glutathione peroxidase), SOD1 and SOD2 (isoforms of superoxide dismutases), Cat (catalase), and Nrf-2 (nuclear factor erythroid-derived 2-like 2), and the NOX1 and NOX4 (NADPH oxidases) expression decreased, which is a sign of increased cellular antioxidant status. At the same time, there was an increase in the expression of the Mao-A and Mao-B genes (monoamine oxidases), which, on the one hand, contribute to the oxidation of a number of transmitters and their availability as a substrate for energy production, but on the other hand, are capable to producing ROS (Figure 5A). In the chronic GluTox model, cell pre-incubation with 50 μg/mL SELENOM led to a decrease in the TXNRD2, TXNRD3, DIO1, GPX1, GPX3, and Cat (catalase) gene expression, which is a sign of antioxidant protection depletion by exogenous SELENOM. However, there were also positive effects of recombinant SELENOM on the cellular redox status during glutamate excitotoxicity when there was an increase SOD2 expression and a NOX1 decrease (Figure 5B). In the OGD/R model, similar trends in gene expression were observed after 24 h pre-incubation of cells with 50 μg/mL recombinant SELENOM. After OGD/R, pre-incubation with recombinant SELENOM led to a decrease in TXNRD3, GPX1, HO-1 (heme oxygenase), Cat, and NRF-2 genes, which may have been a sign of depletion of the cell antioxidant system. But along with these changes, there was a NOX1, NOX4, and Mao-A decrease and an increase in SOD1 and SOD2 expression, which contributes to protection from ROS generated during OGD/R (Figure 5C).

**Figure 6:**
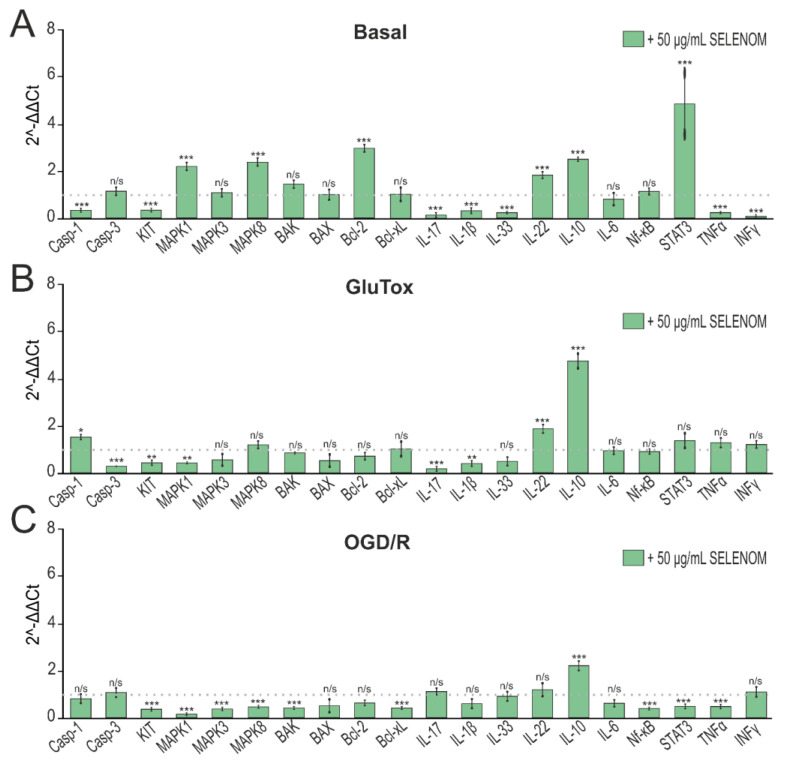
Effect of 24 h pre-incubation of cortical neuroglial cultures with 50 μg/mL exogenous SELENOM on basal (**A**), GluTox-induced (**B**), and OGD/R-induced (**C**) expression of genes encoding proteins involved in cell death. The dotted line indicates protein expression in cortical cells treated with solvent buffer for panel (**A**), in cells after 24 h of GluTox exposure without pre-incubation with SELENOM for panel (**B**), and ischemia-like conditions (OGD/R) without pre-incubation with SELENOM for panel (**C**). Results are presented as mean ± SEM. Black asterisks indicate the differences between the experimental groups comparable to the Buffer-treated group (**A**), GluTox-treated group (**B**), and OGD/R-treated group (**C**) without pre-incubation with SELENOM. n/s—data not significant (*p* > 0.05), *** *p* < 0.001, ** *p* < 0.01, and * *p* < 0.05.

Analysis of gene expression obtained by real-time PCR showed that 24 h pre-incubation of cortical cells with 50 μg/mL of exogenous SELENOM led to a decrease in pro-inflammatory gene expression. In particular, one can note a downward trend in Casp-1, which is known to initiate the inflammatory response through the cleavage and activation of IL-1β, the expression of which was also decreased. In addition, a decrease in the expression of mRNA of other pro-inflammatory cytokines IL-17, IL-33, TNFα, and INFγ can be noted. At the same time, the absence of growth of pro-apoptotic genes encoding Casp-3, BAX, and BAK can be detected (Figure 6A). Long-term exposure to excitotoxic doses of glutamate activates nerve cell death pathways. After 24 h pre-incubation of cells with 50 μg/mL of exogenous SELENOM, in general, the effect of glutamate on neuroglial cultures did not significantly affect the mRNA expression patterns of pro-inflammatory and pro-apoptotic genes. On the contrary, a decrease in the expression of Casp-3 and BAX mRNA can be noted (Figure 6B). Cytoprotective effects of exogenous SELENOM were also reported in an ischemia-like condition (OGD/R) model. Pre-incubation of cortical cells with 50 μg/mL of exogenous SELENOM for 24 h protected neuroglial cells from the negative effects of oxygen and glucose deprivation/reoxygenation, increasing the expression of anti-inflammatory genes and reducing a number of pro-inflammatory interleukins and Casp-1. It should also be noted that SELENOM induced a reduction in the expression of MAPK1, MAPK3, and MAPK8 genes, which also promoted the survival of cortical cells in OGD/R (Figure 6C).

Thus, exogenous recombinant SELENOM showed cytoprotective efficacy in two cellular toxic models. Recombinant SELENOM dose-dependently suppressed brain cell necrosis under the excitotoxic effect of glutamate and ischemia-like conditions. Pre-incubation of cortical cells with recombinant SELENOM contributed to an increase in the antioxidant status of cells; however, after exposure to excitotoxic doses of glutamate or ischemia-like conditions, on the contrary, recombinant SELENOM potentiated the depletion of the cellular antioxidant system by suppressing the expression of mainly selenium-containing proteins on the one hand, but on the other hand, an increase in the expression of key antioxidant genes SOD1 and SOD2 and a decrease in the expression of NOX1 and NOX4 were observed. In addition to the effects on the redox status, exogenous SELENOM showed itself to be a powerful anti-inflammatory protein, reducing the expression level of almost all the studied genes encoding pro-inflammatory cytokines and factors. At the same time, this protective effect is preserved in the glutamate excitotoxicity model, and in the case of ischemia-like conditions, recombinant SELENOM showed the most pronounced protective effect, recorded by gene expression.

### 3.2. The Effect of Different Concentrations of Exogenous SELENOM on the Redox Status of Cortical Cells and the Production of Reactive Oxygen Species

**Figure 7:**
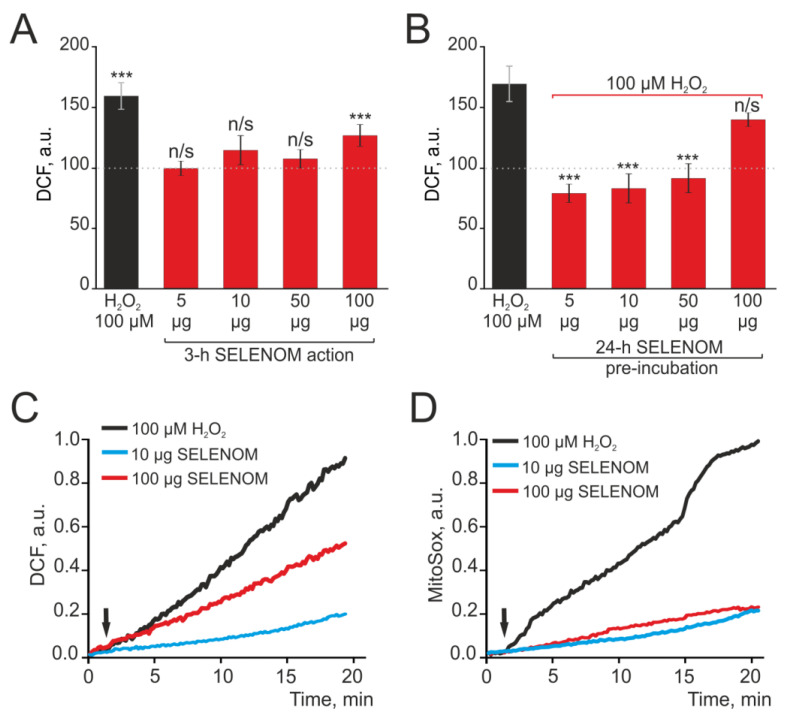
Dose-dependent effect of acute (**A**,**C**,**D**) recombinant SELENOM action and 24 h pre-incubation (**B**) of cortical astrocytes with different concentrations of SELENOM on ROS production. (**A**) ROS production in cortical astrocytes on the application of various SELENOM concentrations in an acute experiment after 3 h of recording DCF fluorescence. (**B**) ROS production in cortical astrocytes after 24 h pre-incubation with various SELENOM concentrations. Data obtained with an automated multiplate reader (Spark™ 10M multimode microplate reader) are presented. Data are shown as the mean of fluorescence intensity, arb.units ± S.E.M. Statistical analysis of experimental groups versus Control (dashed line) was performed with a paired *t*-test. *** *p* < 0.001, and n/s—insignificant differences (*p* > 0.05). (**C**,**D**) Predominantly cytosolic ROS production (**C**) and mitochondrial ROS production (**D**) in cortical astrocytes in response to different concentrations of SELENOM and 100 μM H_2_O_2_. The curves of ROS production averaged over several dozens of cells are presented.

A number of proteins have antioxidant functions in cells. The antioxidant effect of peroxiredoxins, including recombinant forms added to cells or injected into the body, has been well demonstrated [29,30,31]. In cells, recombinant SELENOM performs an antioxidant function, and the effects of exogenous action of recombinant SELENOM have not been studied. Mouse cortical astrocytes were seeded into 96-well plates and, after reaching confluency, were loaded with the DCF-DA probe. Various concentrations of exogenous SELENOM were added to the wells of the plate, and ROS production was recorded for 3 h using a microplate reader. Figure 7A shows that after 3 h of exposure to 5 μg, 10 μg, or 50 μg recombinant SELENOM, there was no increase in ROS production by astrocytes, but 100 μg of protein led to a 32% increase in ROS production relative to the control (Figure 7A—dotted line). ROS production under the influence of 100 μg recombinant SELENOM was found to be comparable to H_2_O_2_-induced ROS production in astrocytes (Figure 7A—black column).

Pre-incubation of cortical astrocytes with various concentrations of exogenous SELENOM for 24 h followed by the application of 100 μM H_2_O_2_ led to the induction of protective effects of this protein. After 24 h exposure with 1 μg, 10 μg, and 50 μg recombinant SELENOM, the application of 100 μM hydroperoxide did not cause ROS production, and this parameter remained at the level of control cells (Figure 7B). After incubating astrocytes for 24 h with 100 μg recombinant SELENOM, no such suppression was observed, and H_2_O_2_ caused a marked increase in ROS production (Figure 7B).

To identify the source of ROS production under the exogenous SELENOM action, cortical astrocytes were grown on round coverslips and loaded with DCF-DA probes, the appearance of fluorescence of which primarily reflects the production of ROS by cytosolic enzymes or the MitoSox Red probe, which allows for the recording of ROS production by mitochondria. When astrocytes were exposed to 100 μM H_2_O_2_ in an acute experiment, ROS production was recorded both in the cytosol (Figure 7C—black curve) and in mitochondria (Figure 7D—black curve). Application of 10 μg SELENOM to astrocytes caused comparable weak ROS production both in the cytosol (Figure 7C—blue curve) and in mitochondria (Figure 7D—blue curve). However, upon application of 100 μg recombinant SELENOM, increased ROS production was recorded mainly in the cytosol of astrocytes (Figure 7C—red curve), while MitoSox Red fluorescence in the mitochondria (Figure 7D—red curve) was at the concentration level of 10 μg recombinant SELENOM.

**Figure 8:**
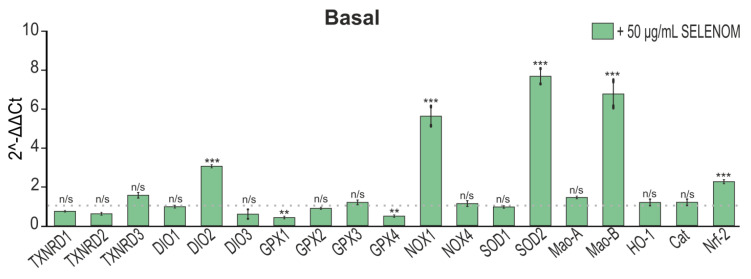
The effect of 24 h pre-incubation of astrocytes from a pure cortical culture with 50 μg/mL recombinant SELENOM on the level of gene expression encoding redox status proteins. The dotted line indicates expression in cortical astrocytes treated with protein solvent buffer. The results are presented as mean ± SEM. Black asterisks indicate the differences between the experimental groups comparable to the Buffer-treated group. n/s—data not significant (*p* > 0.05), *** *p* < 0.001, ** *p* < 0.01.

Analysis of the gene-expression-encoding proteins of redox status in cortical astrocytes after 24 h exposure to 50 μg/mL recombinant SELENOM showed that there was an increase in the expression of the SOD2, DIO2, and Nrf-2 genes encoding antioxidant enzymes; however, an increase in gene expression was also observed NOX1 and Mao-B, capable of producing ROS under certain conditions (Figure 8). At the same time, there was a decrease in the expression of the GPX1 and GPX4 genes, which indicates some depletion of the antioxidant system of astrocytes when exposed to recombinant SELENOM (Figure 8).

Thus, exogenous recombinant SELENOM in high concentrations was found to be capable of inducing ROS production in astrocytes comparable to H_2_O_2_-induced ROS production. The source of the increase in ROS in astrocytes under the influence of SELENOM was cytosolic enzymes. SELENOM concentrations of 5–50 μg did not cause an increase in ROS production after a 3 h exposure, but after a 24 h pre-incubation, they were able to have an antioxidant effect and suppress H_2_O_2_-induced ROS production. Analysis of the expression of genes encoding redox status proteins confirmed that SELENOM is able to influence the redox status of astrocytes and ROS production by changing genome expression patterns.

### 3.3. Exogenous SELENOM Inhibited the Global [Ca^2+^]_i_ Increase and Cell Death in Cortical Cells under Glutamate Excitotoxicity (GluTox) and Ischemia-like Conditions (OGD) In Vitro

**Figure 9:**
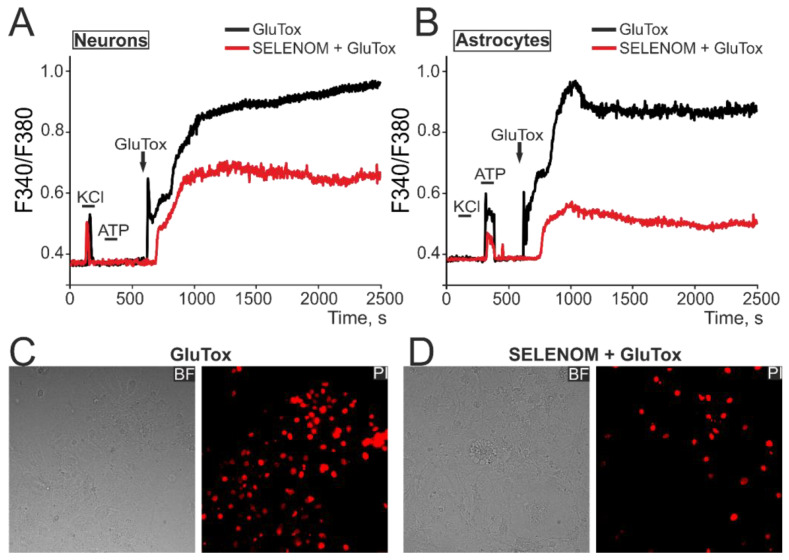
Effect of exogenous SELENOM on Ca^2+^ signals of neurons (**A**) and astrocytes (**B**) and their death in a mixed cortical neuroglial culture when exposed to glutamate excitotoxicity (GluTox) for ~40 min. (**A**,**B**) Ca^2+^ signals of neurons (**A**) and astrocytes (**B**) under GluTox, depending on pre-incubation for 24 h with 50 µg/mL SELENOM. The Ca^2+^ signals of neurons and astrocytes averaged over several dozen cells in one experiment are presented. Neurons were distinguished from astrocytes using a short-term (30 s) application of 35 mM KCl, to which only neurons responded by generating Ca^2+^ signals. Conversely, astrocytes responded with Ca^2+^ signals to the addition of 10 µM ATP. (**C**,**D**) Propidium iodide (PI) staining of cell cultures after ~40 min of GluTox without pre-incubation with SELENOM (**C**) and after 24 h of pre-incubation with SELENOM (50 µg/mL). The appearance of PI fluorescence (red cell nuclei) reflects necrotic cell death.

To simulate glutamate excitotoxicity (GluTox) in an acute experiment, cortical neuroglial cultures were supplemented with 100 µM glutamate in a magnesium-free medium supplemented with 20 µM glycine. In response to GluTox, a rapid biphasic increase in [Ca^2+^]_i_ was recorded in neurons (Figure 9A—black curve) and in astrocytes (Figure 9B—black curve), characterized by a rapid first phase of hyperexcitation and a second phase of global [Ca^2+^]_i_ increase. Staining of cells after ~40 min of GluTox with propidium iodide (PI) showed the appearance of dead cells (Figure 9C—GluTox), detected by the disruption of the cell membrane integrity and the appearance of PI fluorescence. After 24 h pre-incubation of cells with 50 µg/mL exogenous SELENOM, the first phase of the GluTox-induced [Ca^2+^]_i_ increase was inhibited and the global [Ca^2+^]_i_ increase in neurons was significantly suppressed (Figure 9A—red curve). However, the most pronounced inhibitory effect of exogenous SELENOM was detected in cortical astrocytes, in which a more powerful suppression of the GluTox-induced [Ca^2+^]_i_ increase occurred (Figure 9B—red curve). Although pre-incubation of cortical cells with exogenous SELENOM did not completely suppress the global [Ca^2+^]_i_ increase under the excitotoxic effect of glutamate, there was a suppression of cell death, as confirmed by a decrease in PI-positive cells (Figure 9D).

**Figure 10:**
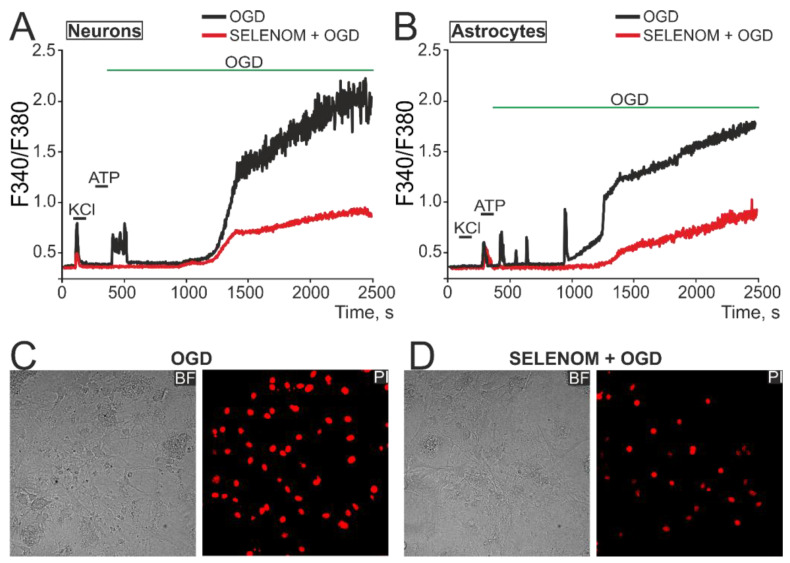
Effect of exogenous SELENOM on Ca^2+^ signals of neurons (**A**) and astrocytes (**B**) and their death after ischemia-like conditions (OGD, oxygen-glucose deprivation) for ~40 min. (**A**,**B**) Ca^2+^ signals of neurons (**A**) and astrocytes (**B**) under the OGD, depending on pre-incubation for 24 h with 50 µg/mL SELENOM. The Ca^2+^ signals of neurons and astrocytes averaged over several dozen cells in one experiment are presented. Neurons were distinguished from astrocytes using a short-term (30 s) application of 35 mM KCl, to which only neurons responded by generating Ca^2+^ signals. Conversely, astrocytes responded with Ca^2+^ signals to the application of 10 µM ATP. (**C**,**D**) Propidium iodide (PI) staining of cell cultures after ~40 min OGD without pre-incubation with SELENOM (**C**) and after 24 h pre-incubation with SELENOM (50 µg/mL). The appearance of PI fluorescence (red cell nuclei) reflects necrotic cell death.

Simulation of acute ischemia-like conditions (OGD, 40 min) caused a biphasic [Ca^2+^]_i_ increase in neurons (Figure 10A—black curve) and astrocytes (Figure 10B—black curve), when [Ca^2+^]_i_ during the first phase signaling could return to the initial level, but the second phase of Ca^2+^ responses represented a global [Ca^2+^]_i_ increase. After 24 h pre-incubation of cells with 50 µg/mL of exogenous SELENOM, comparable inhibition of the first phase of the OGD-induced [Ca^2+^]_i_ increase and significant suppression of irreversible [Ca^2+^]_i_ increase during the second phase of signaling occurred in neurons (Figure 10A—red curve) and astrocytes (Figure 10B—red curve). As a result, staining of cell cultures after OGD with propidium iodide showed a decrease in the number of necrotic cells (Figure 10D), compared with the effect of OGD without pre-incubation with recombinant SELENOM (Figure 10C).

Thus, pre-incubation of cortical neuroglial cultures for 24 h with recombinant SELENOM can significantly limit the global [Ca^2+^]_i_ increase induced by ischemia-like conditions or the excitotoxic effect of glutamate, which ultimately leads to a decrease in necrotic death of a large number of cells in the culture, but no complete suppression of cell death was detected.

### 3.4. Acute Effects of Exogenous SELENOM on Cortical Cells: Signal Transduction Pathways

**Figure 11:**
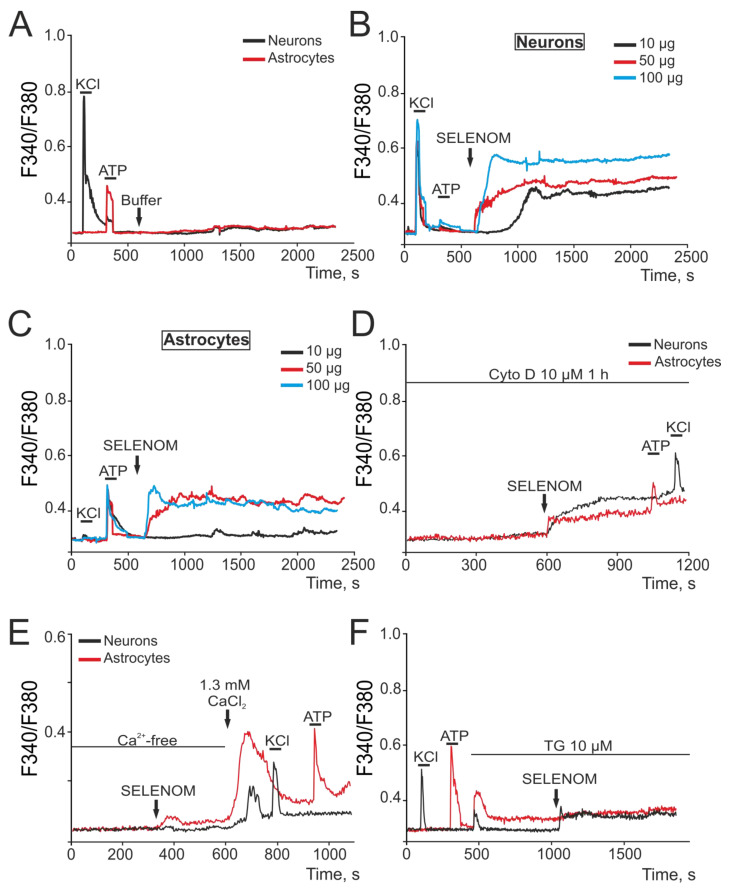
Putative signaling pathways of recombinant SELENOM-induced [Ca^2+^]_i_ increase in cortical neurons and astrocytes. (**A**) Application of 100 μL solvent buffer for SELENOM did not cause Ca^2+^ signal generation in neurons (black curve) and astrocytes (red curve). (**B**,**C**) Application of various exogenous SELENOM concentrations to neurons (**B**) and astrocytes (**C**) in a cortical neuroglial culture. (**D**) Application of 50 μg exogenous SELENOM to a cortical neuroglial culture after 1 h pre-incubation with 10 μM of the actin-dependent endocytosis blocker Cytochalasin D (Cyto D). (**E**) Application of 50 μg exogenous SELENOM to a neuroglial culture in a nominally calcium-free medium (Ca^2+^-free) with the addition of 0.5 mM of the calcium chelator EGTA. (**F**) Application of 50 μg exogenous SELENOM to a neuroglial culture after depletion of the Ca^2+^ store of the endoplasmic reticulum using the SERCA blocker thapsigargin (TG, 10 μM).

Signaling pathways for survival or cell death are closely related and regulated by [Ca^2+^]_i_ changes in brain cells. At this stage, the effects of various exogenous SELENOM concentrations in an acute experiment were studied. To do this, cells were loaded with the calcium-sensitive probe Fura-2. Application of a buffer in which recombinant SELENOM was dissolved in a volume (150 μL) corresponding to the maximum added concentration of recombinant SELENOM (100 μg) did not cause the Ca^2+^ signal generation in cortical neurons and astrocytes of mixed neuroglial culture (Figure 11A). This experiment indicates that there is no artifactual effect of the protein solvent on the cortical cells. Application of SELENOM at 10, 50, and 100 μg concentrations caused the Ca^2+^ signal generation in neurons (Figure 11B) and astrocytes (Figure 11C) of the neuroglial culture. These Ca^2+^-signals were characterized by a primary transient [Ca^2+^]_i_ increase in both the case of 50 μg and 100 μg, wherein there was a gradual slow [Ca^2+^]_i_ increase reaching a new stationary [Ca^2+^]_i_ level. In neurons (Figure 11B), the application of protein dose-dependently caused a rapid [Ca^2+^]_i_ increase and the establishment of a new steady-state level, even at a 10 μg SELENOM concentration. Experiments with recombinant SELENOM applications in a culture of pure cortical astrocytes showed that in the absence of neurons in the culture, astrocytes respond with neuron-like Ca^2+^ signals to the application of this protein (not shown). It can be assumed that differences in the Ca^2+^ signals of neurons and astrocytes in a mixed neuroglial culture are due to neuron–glial interactions, and in the absence of one of the network components, Ca^2+^ signals acquire similar kinetics. However, the explanation for such effects of SELENOM requires further research. It is interesting that exogenous SELENOM is a unique protein of its kind, since its effects on the Ca^2+^ signaling system of brain cells are recorded in an acute experiment. For example, the application of another selenoprotein SELENOV, which is expressed in the cytoplasm and nucleus, did not cause the Ca^2+^ signal generation either in neurons (Figure S2A) or in astrocytes (Figure S2B), while the subsequent application of recombinant SELENOM led to Ca^2+^ signal generation. Similarly, the antioxidant protein peroxiredoxin-6 (Prx-6), which has shown cytoprotective effects in various pathological processes [31,32], did not cause rapid [Ca^2+^]_i_ increases in cortical cells upon application (Figure S2A,B). At the same time, endocytosis mechanisms were involved in the Ca^2+^ signal generation by neurons and astrocytes upon the exogenous SELENOM application, when the cellular Ca^2+^ signals were suppressed by more than 80% after incubation with cytochalasin D (a blocker of actin-dependent transport), and the remaining component of the Ca^2+^ signal was able to occur through SELENOM diffusion since it is a small protein (~16 kDa) (Figure 11D). Ca^2+^ signals in cells develop due by two signaling components—the Ca^2+^ ions mobilization from intracellular pools and the Ca^2+^ ions entry from outside through plasma membrane channels. Application of SELENOM in a calcium-free medium did not cause the Ca^2+^ signal generation in neurons (Figure 11E—black curve), and in astrocytes, there was a significant suppression of the SELENOM-induced Ca^2+^ signal (Figure 11E—red curve). Restoring the Ca^2+^ concentration in the extracellular environment causes an [Ca^2+^]_i_ increase in cells, the so-called Ca^2+^ paradox [33], and the subsequent application of KCl or ATP also causes Ca^2+^ signal generation, which indicates the functionality of the cellular Ca^2+^ signaling system (Figure 11E). Depletion of the thapsigargin-sensitive Ca^2+^ pool by application of thapsigargin (TG, 10 μM) led to complete suppression of SELENOM-induced Ca^2+^ signals in astrocytes (Figure 11F—red curve), and in neurons, there was a decrease in the amplitude of the [Ca^2+^]_i_ increase (Figure 11F—black curve).

**Figure 12:**
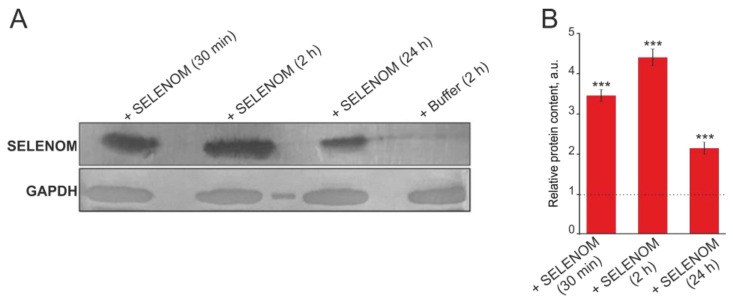
Western blot analysis of cortical cell lysate samples after incubation with 50 μg of exogenous SELENOM or buffer. (**A**) Immunoblotting results obtained using antibodies against hexahistidine-labeled SELENOM at 30 min, 2 h, and 24 h after application of SELENOM to the culture medium. (**B**) Quantification of the studied proteins in the samples obtained using ImageJ software, presented as mean ± standard deviation of three independent experiments. GAPDH was used as a control for normalization. The expression level in the control (without treatment) was taken as 1 (dotted line). Statistical comparisons were made relative to the control group using the *t*-test. *** *p* < 0.001.

Inhibition of actin-dependent transport showed that for the Ca^2+^ signal generation by cortical cells in the recombinant SELENOM application, penetration of the protein into the cells is necessary. Using Western blot analysis with antibodies against polyhistidine tag, we found that recombinant SELENOM is able to accumulate in significant quantities in them within 30 min after application to cells (more than three times compared with the Control). The same trend persisted after 2 h, but after 24 h, the relative content of recombinant SELENOM decreased by approximately half compared to the 2 h pre-incubation (Figure 12A,B).

**Figure 13:**
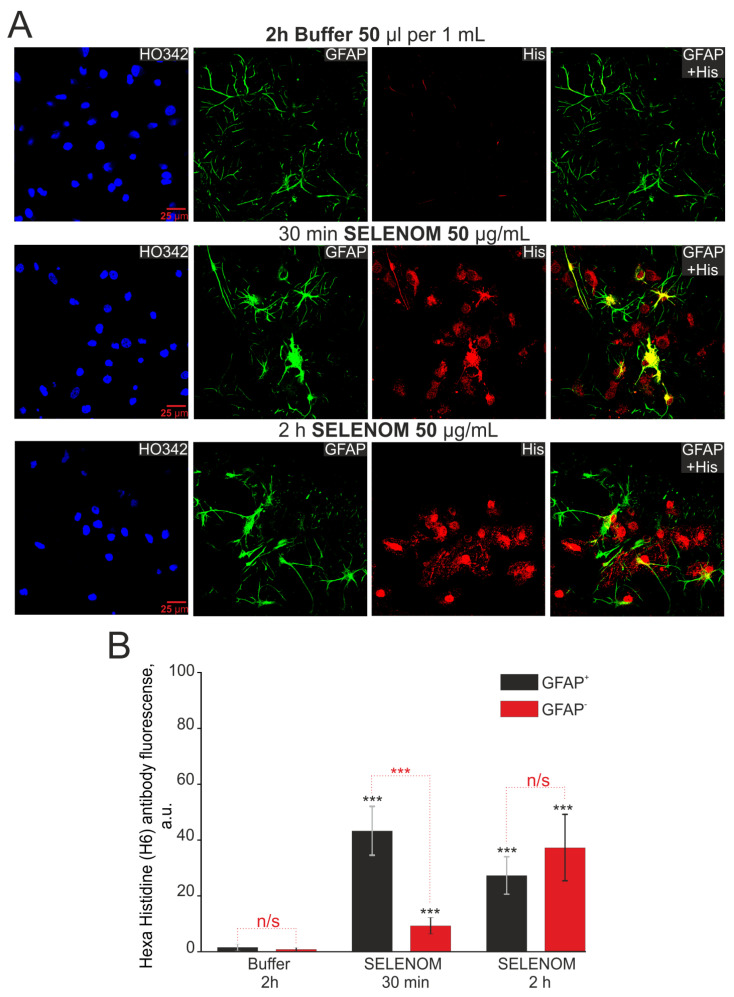
Immunocytochemical staining of cortical cells after pre-incubation with 50 μg/mL recombinant SELENOM for 30 min and 2 h. (**A**) Images of astrocytes (GFAP^+^ cells) and non-astrocytes (GFAP^–^ cells) stained with antibodies against histidine (His), reflecting the presence of recombinant SELENOM in the cells. HO342—cell nuclei stained with Hoechst 33342. GFAP + His—merged images of GFAP^+^ cells and histidine^+^ (His) cells, reflecting the presence of recombinant SELENOM in cortical astrocytes. (**B**) Intensity levels of histidine were determined by confocal imaging. We analyzed individual cells that had fluorescence of secondary antibodies. The quantitative data reflecting the level of histidine expression in GFAP^+^-cells and GFAP^—^-cells are presented as fluorescence intensity values in summary bar charts (mean ± SEM). The values were averaged by 100 cells for each column. The results obtained after immunostaining agree with the data of fluorescence presented in (**A**). Statistical significance was assessed using a paired *t*-test. Comparison with Buffer is marked by black asterisk. Comparisons between experimental groups are marked in red. n/s—data not significant (*p* > 0.05), *** *p* < 0.001.

To determine the specificity of SELENOM accumulation in cortical cells, exogenous SELENOM was applied for 30 min and 2 h, after which the cells were fixed and stained with antibodies against GFAP (an astrocytic marker) and histidine (reflecting the accumulation of recombinant SELENOM) (Figure 13A). After a 2 h pre-incubation of cells with protein solvent buffer, no histidine fluorescence was detected (Figure 13A,B). After 30 min of cell pre-incubation with recombinant SELENOM, fluorescence of secondary antibodies to histidine was detected in both GFAP-positive (GFAP^+^, astrocytes) and GFAP-negative cells (GFAP^-^, neurons) (Figure 13A). Recording fluorescence of secondary antibodies and obtaining images of cell cultures were carried out at the same microscope settings and fluorescence analysis, indirectly, but they were able to provide quantitative indicators of the histidine levels (and therefore SELENOM) in cells. Analysis of fluorescence intensity of secondary antibodies showed that after 30 min, more histidine was detected in astrocytes compared to neurons (Figure 13B). Increasing the time of recombinant SELENOM incubation with cells to 2 h led to increased accumulation of histidine in neurons (GFAP^-^ cells), and in astrocytes, the level of histidine was comparable to a 30 min incubation (Figure 13B). Simultaneous staining of cortical cells with antibodies against histidine and NeuN (a marker of neurons) showed similar effects of exogenous SELENOM. After 30 min cell incubation with recombinant SELENOM, the most pronounced accumulation of histidine was observed in NeuN^−^ cells (astrocytes), and after 2 h of incubation, neurons accumulated comparable amounts of histidine (Figure S3A,B).

Thus, exogenous SELENOM caused rapid activation of the Ca^2+^ signaling system of cortical neurons and astrocytes in a wide concentration range (10–100 μg/mL). Application of recombinant SELENOM caused the Ca^2+^ signal generation with rapid [Ca^2+^]_i_ increase when using a protein concentration of tens of micrograms, followed by the establishment of a new [Ca^2+^]_i_ stationary state at an elevated level that, however, did not cause damage or cell death. The cellular Ca^2+^ signal generation occurred primarily due to Ca^2+^ ion mobilization from the thapsigargin-sensitive pool of the endoplasmic reticulum, especially in astrocytes. Conversely, in neurons, the external Ca^2+^ ion entry is important and possibly key, since in a calcium-free medium, the neuronal Ca^2+^ responses to recombinant SELENOM application were suppressed, and when the ER was depleted, reduced Ca^2+^ signals remained. For the Ca^2+^ signal generation by cortical cells upon recombinant SELENOM application, the penetration of the protein into the cells was necessary, since cell signals were significantly suppressed when endocytosis pathways were inhibited. However, some component of the Ca^2+^ signal, especially in neurons, was preserved after the blocker of actin-dependent endocytosis, cytocholasin D, which can be determined both by the diffusion of the protein into the cells and by some of its effects outside the cells, which requires further study and is not shown in this investigation. Using Western blotting and immunocytochemical staining, it was possible to confirm that recombinant SELENOM quickly penetrates into cells and, after 30 min of exposure, accumulates in large quantities in astrocytes, and after 2 h of pre-incubation, the level of recombinant SELENOM in neurons becomes comparable to astrocytes.

## 4. Discussion

SELENOM is an endoplasmic reticulum resident oxidoreductase, structurally similar to thioredoxin, since it contains a thioredoxin fold in its structure [34]. Thioredoxin is known to catalyze the formation of disulfide bonds through thiol–disulfide exchange, in which the CXXC motif (where C is cysteine and X is any amino acid) plays a key role [35]. In SELENOM, the CXXC motif is found as CXXU, where U refers to Sec [13]. It is assumed that such a replacement of C with U accelerates the reactions of thiol-like disulfide exchange. However, there is relatively little information about the specific processes in which SELENOM, which has structural features similar to thioredoxin, is involved, and the molecular mechanisms of regulation of these processes are also not completely clear. It is known that thioredoxin-interacting protein (TXNIP), also known as thioredoxin-binding protein 2, is a major mediator in the TXN antioxidant system. TXNIP interacts with the CXXC motif of TXN, blocking its potential to scavenge ROS. This in turn increases the cellular ROS levels and induces apoptosis [10]. Micro-array analyses of SELENOM-defective mHypoE-44 cells and hypothalamic tissue identified a downregulation of TXNIP but no change in expression levels of TXN and TXNRD [20]. In the absence of SELENOM, brain tissue experienced increased ER stress, apoptosis, and inflammation, as well as increased TXNIP levels [36,37]. That is, in the case of SELENOM deletion, a decrease in TXNIP may be a compensatory mechanism for the absence of SELENOM. Our studies showed that pre-incubation of cortical cells with various concentrations of exogenous SELENOM led to a significant inhibition of cell death in two toxic models—glutamate excitotoxicity and ischemia/reoxygenation. It should be said that there was no complete inhibition of cell death, but inflammatory processes (necrosis) and the late stages of apoptosis were suppressed. At the same time, cells that were at an early (reversible) stage of apoptosis were preserved. It is known that controlled cell death is the more preferable method for the brain cells death during pathological processes [38]. Oxidative stress is a key contributor to brain cell death during injury, especially ischemia [39]. As our studies showed, exogenous SELENOM in concentrations up to 50 μg/mL did not cause ROS production by mitochondria or cytosolic enzymes within 3 h of recording, but the application of 100 μg/mL SELENOM led to ROS production predominantly by cytosolic enzymes. At the same time, pre-incubation of cortical astrocytes with 5–50 μg/mL recombinant SELENOM for 24 h correlated with the suppression of ROS production upon the application of hydroperoxide. However, the use of 100 μg/mL recombinant SELENOM, on the contrary, potentiated H_2_O_2_-induced ROS production. The fact that the effects of SELENOM depend on protein concentration and exposure time should be taken into account when studying this and similar proteins. It is known that recombinant SELENOM is involved in the regulation of the cellular redox status. Knockout of SELENOM has been shown to affect mitochondrial ROS production. Without stress treatments, basal ROS production is similar in the cells of neuronal origin, but under ER stress, selenium-depleted cells show increased mitochondrial ROS production and death due to oxidative stress [20].

Since glutathione is a key defense system in the brain against oxidative stress, there is evidence that selenium suppresses glutathione depletion [40]. SELENOM is an endoplasmic reticulum resident protein and is expressed at high levels in the brain compared to other tissues [41]. For brain cells (HT22 hippocampal cells and C8-D1A cerebellum cells), it was found that SELENOM modulates the Ca^2+^ ion mobilization from the ER under H_2_O_2_-induced oxidative stress. Overexpression of SELENOM in HT22 cells and primary cortical cells protected against H_2_O_2_-induced oxidative stress, and knockdown of SELENOM increased apoptosis in these cells [19,42]. In addition, SELENOM can bind metal ions such as Zn^2+^ and limit amyloid-beta aggregation, suppress ROS production, and reduce amyloid neurotoxicity in Alzheimer’s disease [43]. Our results correlate well with the above studies of other investigators, since pre-incubation of cortical cells with exogenous SELENOM reduced cell death and inhibited ROS production under glutamate excitotoxicity, ischemia/reoxygenation, and hydroperoxide. The neuroprotective effects of SELENOM may be mediated by Galectin-1 [44]. Using transgenic CMV/hSelM Tg rats, it was found that overexpression of SELENOM and consumption of selenium sources by animals led to an increase in the brain antioxidant system and changes in the expression of nine genes contributing to selenium bioavailability in the brain. Among these genes, especially noteworthy is the gene encoding synaptotagmin-15, which is an intracellular Ca^2+^ sensor that regulates Ca^2+^-dependent membrane trafficking processes, including endocrine exocytosis, synaptic vesicle exocytosis, and neurotransmitter release [45,46].

Our work examined the expression of mRNA of a wide range of genes encoding selenium-containing oxidoreductases, selenoproteins with poorly studied functions, antioxidant enzymes, anti- and pro-inflammatory interleukins, etc. It is important to note that the effect of recombinant SELENOM on cortical cells at a concentration of 50 μg/mL for 24 h contributed to the activation of a number of antioxidant enzymes, such as superoxide dismutases 1 and 2, catalase, and monoamine oxidases A and B. There was also a tendency towards an increase in the expression of key selenium-containing oxidoreductases of the thioredoxin and glutaredoxin systems; TXNRD2; and GPX1, -2, and -4. In addition, pro-apoptotic and pro-inflammatory markers decreased, and a simultaneous increase in the anti-inflammatory interleukin mRNA expression was established. In addition, SELENOM contributed to a decrease in NADPH oxidases 1 and 4 mRNA expressions, which is consistent with the physiological conditions under which the expression and activity of these enzymes are known to be maintained at within relatively low levels of constitutive activity that regulates redox signaling in the vicinity of target molecules [47]. Rats overexpressing SELENOM (CMV/hSelM Tg rats) show increased levels of antioxidant proteins, especially SOD and GPX [45]. SELENOM knockdown mice on a high-calorie diet exhibit liver abnormalities such as oxidative stress, inflammation, steatosis, and fibrosis [48]. Thus, the effect of exogenous SELENOM on cortical cells creates favorable conditions for preventing such negative severe consequences of both glutamate excitotoxicity and ischemia/reoxygenation, restraining strong fluctuations in the expression of mRNA of key enzymes involved in maintaining redox homeostasis and the development of inflammatory reactions, as well as apoptotic and necrotic cell death.

Cortical neurons overexpressing SELENOM are characterized by reduced Ca^2+^ signals to H_2_O_2_-induced oxidative stress. It is known that oxidative stress involves the Ca^2+^ ion mobilization from the ER. Incubation of neurons with H_2_O_2_ in a calcium-free medium with the addition of the Ca^2+^ chelator EGTA did not abolish Ca^2+^ signals, but it reduced their amplitude, which indicates the leading role of the ER in the induction of oxidative stress. Mobilization of Ca^2+^ ions from the ER leads to an Ca^2+^ increase in the cytosol and redistribution of Ca^2+^ ions in mitochondria, which enhances the ROS production. SELENOM overexpression in neurons reduced the Ca^2+^ signal to stimuli that mobilize Ca^2+^ ions from the ER; as a result, there was no increase in mitochondrial ROS production [19]. The correlation between the ROS production, the excitotoxic effect of glutamate, the disruption of ion homeostasis, and the nerve cells death during ischemia is well known [49,50]. Thus, our studies established that 24 h pre-incubation of neuroglial cultures with SELENOM led to the suppression of the OGD-induced global [Ca^2+^]_i_ increase in an acute experiment and a decrease in the number of necrotic cells. It turned out that exogenous SELENOM is able to activate the Ca^2+^ signaling system of neurons and astrocytes in an acute experiment. An [Ca^2+^]_i_ increase upon the application of SELENOM to neuroglial cultures occurred due to the penetration of the protein into the cells through predominantly actin-dependent transport pathways and partly due to diffusion. On the contrary, the application of other proteins, for example, SELENOV or peroxiredoxin-6, which has antioxidant and cytoprotective effects [51], did not cause the Ca^2+^ signal generation in cells, which shows the special uniqueness of SELENOM. SELENOV is a selenoprotein that is expressed in the nucleus and cytoplasm and, like SELENOM, has glutathione peroxidase and thioredoxin reductase activity [52]. Ca^2+^ signals upon SELENOM application occurred in astrocytes due to Ca^2+^ mobilization from the thapsigargin-sensitive pool of the endoplasmic reticulum, and in neurons, SELENOM-induced signaling strongly depended on the external Ca^2+^ ion concentration. It is well known that a moderate or transient [Ca^2+^]_i_ increase activates the expression of defense proteins, while a global uncontrolled [Ca^2+^]_i_ increase causes cell death [53,54,55]. Glutamate excitotoxicity (GluTox) is one of the damaging factors of ischemia [56]. Ischemia-like conditions (OGD) are always accompanied by excessive glutamate release, activation of glutamate receptors, and [Ca^2+^]_i_ increase, so we considered the effects of SELENOM in the GluTox and OGD models together. Conversely, OGD/R (oxygen-glucose deprivation with reoxygenation) is an even more damaging model for studying neurodegeneration in vitro [56,57], since an additional damaging factor is increased oxidative stress during reoxygenation. In our experiments, exogenous SELENOM dose-dependently caused a moderate [Ca^2+^]_i_ increase and the formation of a new steady state for the cellular Ca^2+^ signaling system, which correlated with the cellular protection from death under GluTox or OGD/R, which should be considered as a cytoprotective effect.

The presented study of the exogenous SELENOM effects on cortical cells is a pilot study. Using a complex of modern methods, we were able to identify several important effects that recombinant SELENOM has on cortical cells (Figure S4), and most of them can be defined as positive. Many of the effects of recombinant SELENOM on cortical cells were demonstrated for the first time. However, the presented study is not without a number of limitations. First of all, all results were obtained with in vitro models, and despite the use of two toxic models (GluTox and OGD/R), the data need to be confirmed by in vivo models. In addition, we found that recombinant SELENOM penetrates the plasma membrane and accumulates in cortical cells, thereby exerting cytoprotective effects against toxic influences. At the same time, when using SELENOM concentrations in the 5–50 μg range, no ROS generation occurred, while the use of 100 μg of recombinant protein induced ROS production. Thus, the challenge is to study the effects of high doses of SELENOM and the associated time of the protein action. In this work, pre-incubation of cells for more than 24 h was not studied, although it can be assumed that there are negative effects of the accumulation of SELENOM in the cells in excessive quantities.

## 5. Conclusions

Thus, at this stage of research, a construct for producing recombinant SELENOM in a bacterial system was obtained, and a method for its purification was developed, which made it possible to demonstrate the cytoprotective effect of this protein when modeling glutamate excitotoxicity and ischemia/reoxygenation in cerebral cortical cells. Using PCR analysis, it was possible to establish the selective targeting of exogenous SELENOM on the expression of genes encoding redox status regulators and anti-inflammatory cytokine proteins. The cytoprotective effect of exogenous recombinant SELENOM involves activation of the Ca^2+^ signaling system of neurons and astrocytes in the form of a moderate [Ca^2+^]_i_ increase and the establishment of a new steady state for Ca^2+^ ions, which correlated with the suppression of cell death.

## Data Availability

The data presented in this study are available on request from the corresponding author.

## Supplementary Materials

The following supporting information can be downloaded at https://www.mdpi.com/article/10.3390/biomedicines12081756/s1. Figure S1: PAGE-electrophoresis of proteins in the soluble fraction, sediment, and purified hSELENOM on nickel agarose. Figure S2: Application of exogenous proteins to cortical neurons and astrocytes. Figure S3: Immunocytochemical staining of cortical cells after preincubation with 50 μg/mL SELENOM for 30 min and 2 h. Figure S4: A flow chart reflecting the complex of methods used in the study and the key results of the study.
