# Supplementary material for: Regulatory Role and Cytoprotective Effects of Exogenous Recombinant SELENOM under Ischemia-like Conditions and Glutamate Excitotoxicity in Cortical Cells In Vitro
Source: Biomedicines. 2024 Aug 5;12(8):1756. doi: 10.3390/biomedicines12081756 (PMC11351740; doi:10.3390/biomedicines12081756)
Supplement: Supplementary file 1 [file biomedicines-12-01756-s001.zip › biomedicines-3091343-supplementary.pdf]

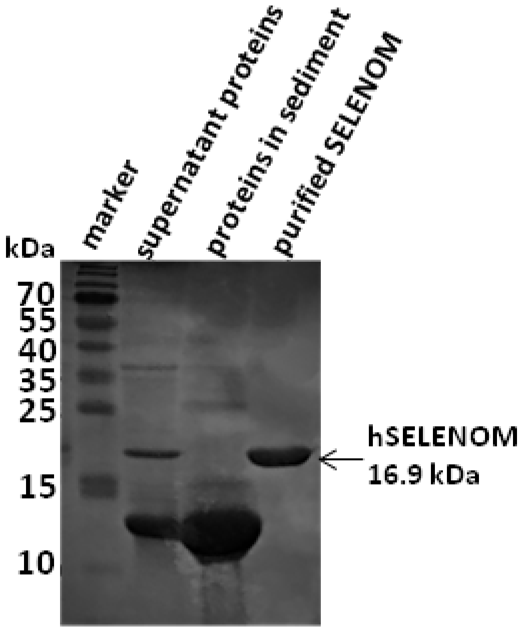

Supplementary, Figure S1. PAGE-electrophoresis of proteins in the soluble fraction, sediment and purified hSELENOM on nickel agarose. PAGE-electrophoresis was performed in 12.5% acrylamide gel. The results of PAGE electrophoresis are presented, visualizing the various stages of SELENOM purification.

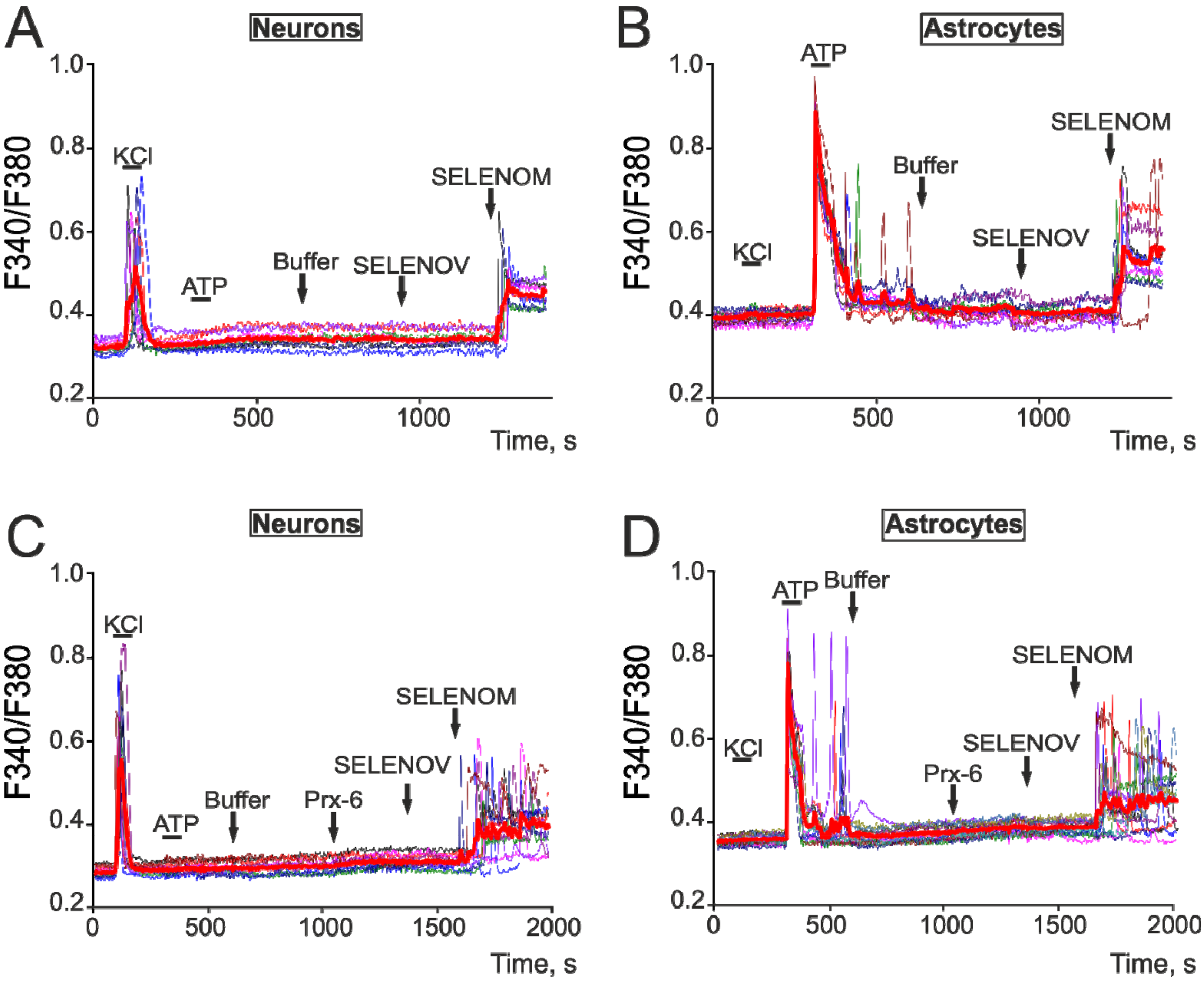

Supplementary, Figure S2. Application of exogenous proteins to cortical neurons and astrocytes. **A**, **B** – Selenoprotein V (SELENOV, 50 μg/mL) and selenoprotein M (SELENOM, 50 μg/mL) application to cortical neurons (**A**) and astrocytes (**B**). **C**, **D** – Application of the antioxidant protein peroxiredoxin-6

(Prx-6, 100 μg/mL), selenoprotein V (SELENOV, 50 μg/mL) and selenoprotein M (SELENOM, 50 μg/mL) to cortical neurons (**C**) and astrocytes (**D**). Buffer – application of an equivalent volume of selenoprotein solvent buffer (50 μl) to the cells. The cellular Ca^{2+} signals in one experiment and their average value are presented (thick red curve). To identify neurons, a short-term application of KCl (35 mM) was performed. Astrocytes were identified by the generation of Ca^{2+} signals upon application of ATP (10 μM).

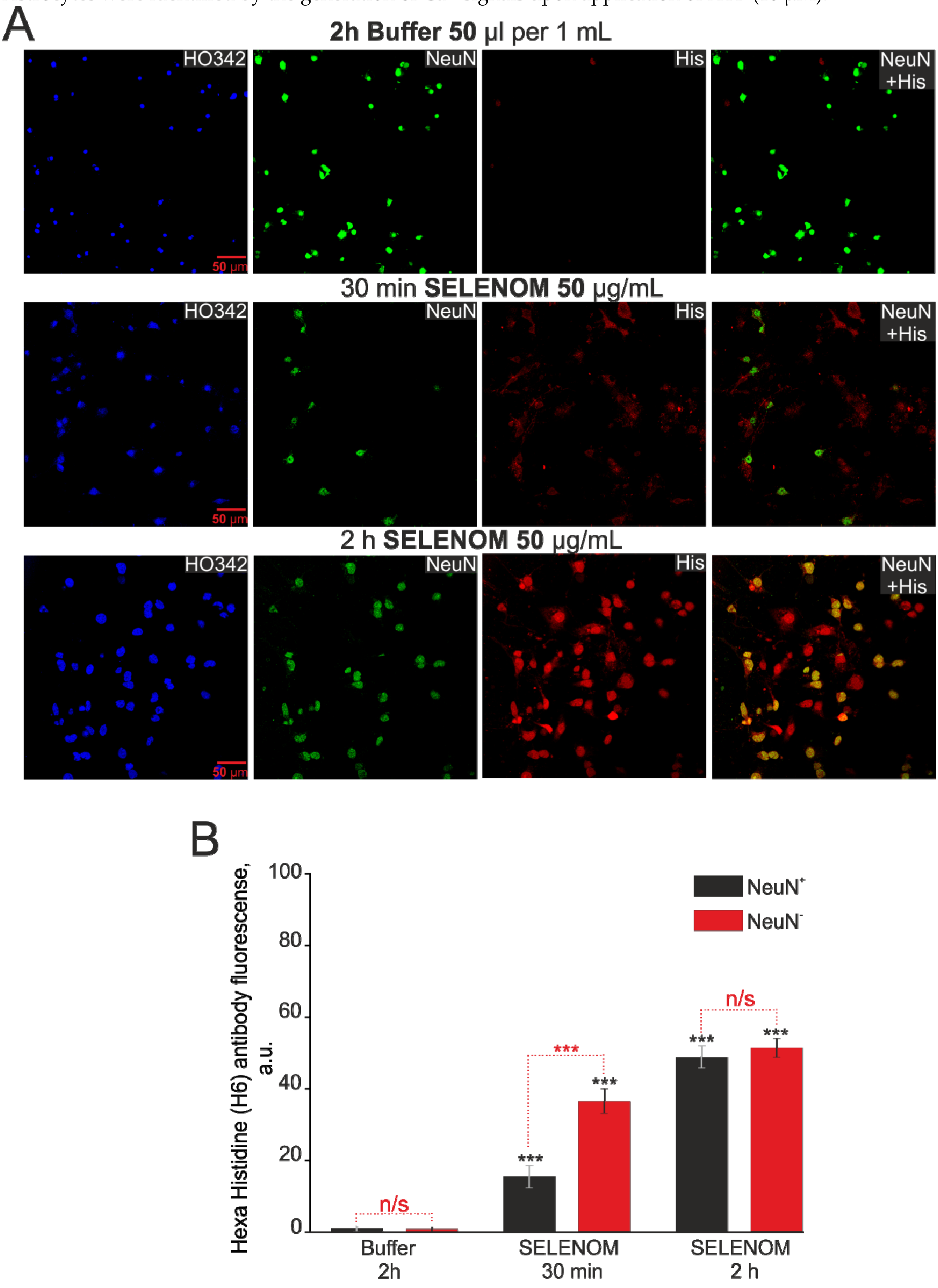

Supplementary, Figure S3. Immunocytochemical staining of cortical cells after preincubation with 50 μg/mL SELENOM for 30 minutes and 2 hours. **A** – Images of neurons ($NeuN^+$-cells) and non-neurons ($NeuN^-$-cells) stained with antibodies against histidine (His), reflecting the presence of SELENOM in the cells. HO342 – cell nuclei stained with Hoechst 33342. NeuN + His – merge images of $NeuN^+$-cells and $histidine^+$ (His^+) cells, reflecting the presence of SELENOM in cortical neurons. **B** - Intensity levels of histidine were determined by confocal imaging. We analyzed individual cells that had fluorescence of secondary antibodies. The quantitative data reflecting the level of histidine expression in $NeuN^+$-cells and $NeuN^-$-cells are presented as fluorescence intensity values in summary bar charts (mean +/- SEM). The values were averaged by 100 cells for each column. The results obtained after immunostaining agree with the data of fluorescence presented in (**A**). Statistical significance was assessed using paired t-test. Comparison with Buffer marked by black asterisk. Comparisons between experimental groups are marked in red. n/s—data not significant ($p > 0.05$), *** $p < 0.001$.

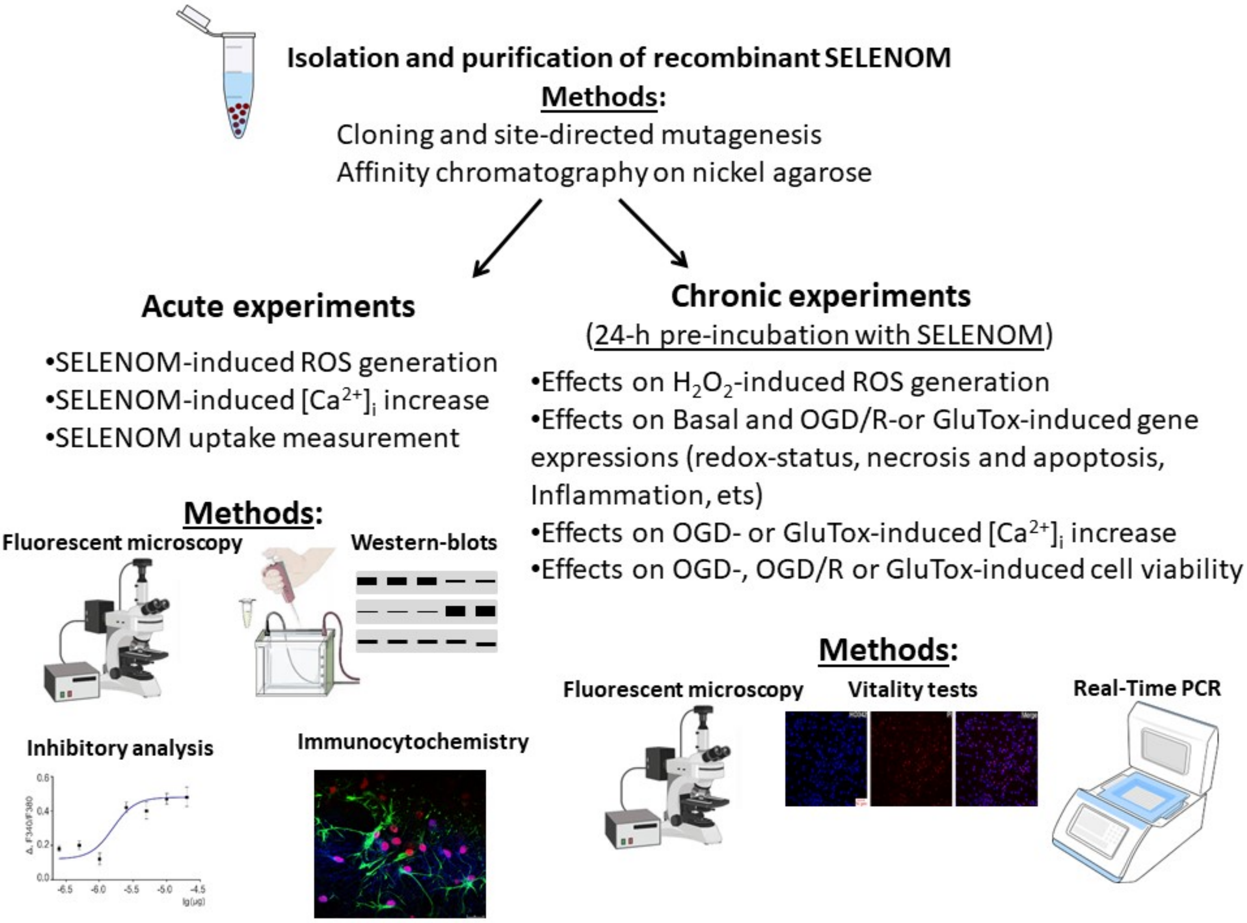

Supplementary, Figure S4. A flow chart reflecting the complex of methods used in the study and the key results of the study.
